# Behavioral Effects of Developmental Exposure to JWH-018 in Wild-Type and Disrupted in Schizophrenia 1 (*disc1*) Mutant Zebrafish

**DOI:** 10.3390/biom11020319

**Published:** 2021-02-19

**Authors:** Judit García-González, Bruno de Quadros, William Havelange, Alistair J. Brock, Caroline H. Brennan

**Affiliations:** 1 School of Biological and Chemical Sciences, Queen Mary, University of London, London E1 4NS, UK; judit@perala.es (J.G.-G.); brunoequadros@hotmail.com (B.d.Q.); w.havelange@qmul.ac.uk (W.H.); 2 Leica Biosystems Newcastle Ltd, Newcastle NE12 8EW, UK; alistair.brock@leicabiosystems.com

**Keywords:** zebrafish, cannabinoids, *disc1*, JWH-018, forced light/dark assay, anxiety

## Abstract

Synthetic cannabinoids can cause acute adverse psychological effects, but the potential impact when exposure happens before birth is unknown. Use of synthetic cannabinoids during pregnancy may affect fetal brain development, and such effects could be moderated by the genetic makeup of an individual. Disrupted in schizophrenia 1 (*DISC1*) is a gene with important roles in neurodevelopment that has been associated with psychiatric disorders in pedigree analyses. Using zebrafish as a model, we investigated (1) the behavioral impact of developmental exposure to 3 μM 1-pentyl-3-(1-naphthoyl)-indole (JWH-018; a common psychoactive synthetic cannabinoid) and (2) whether *disc1* moderates the effects of JWH-018. As altered anxiety responses are seen in several psychiatric disorders, we focused on zebrafish anxiety-like behavior. Zebrafish embryos were exposed to JWH-018 from one to six days post-fertilization. Anxiety-like behavior was assessed using forced light/dark and acoustic startle assays in larvae and novel tank diving in adults. Compared to controls, both acutely and developmentally exposed zebrafish larvae had impaired locomotion during the forced light/dark test, but anxiety levels and response to startle stimuli were unaltered. Adult zebrafish developmentally exposed to JWH-018 spent less time on the bottom of the tank, suggesting decreased anxiety. Loss-of-function in *disc1* increased anxiety-like behavior in the tank diving assay but did not alter sensitivity to JWH-018. Results suggest developmental exposure to JWH-018 has a long-term behavioral impact in zebrafish, which is not moderated by *disc1*.

## 1. Introduction

Synthetic cannabinoids commercialized as “spice”, “K2”, “legal weed”, or “herbal incense” are man-made chemicals used as an alternative to marijuana. They gained popularity during the early 2000s and were legal in many countries [1], with consumption prevalence ranging between 0.2% and 4% in the general population [2]. Among pregnant women, cannabis use has risen in recent years [3], and a similar trend may be true for synthetic cannabinoids, but, for the latter, prevalence estimates in pregnant women are unavailable. 

The endocannabinoid system plays an important role in early brain development [4]; thus, consumption of cannabinoids (both natural or synthetic) during pregnancy may affect fetal neurodevelopment, leading to long-term behavioral alterations [5]. Delta-9-tetrahydrocannabinol (THC), the major psychoactive component of cannabis, can cross the placental barrier [5], bind the cannabinoid receptors located in the fetus brain, and interfere with the endocannabinoid system affecting neurogenesis and neuronal migration [5]. Stronger effects on brain development may be produced by JWH-018 (1-pentyl-3-(1-naphthoyl)-indole), one of the most common psychoactives in synthetic cannabinoids. Behavioral measures in monkeys and rats suggest JWH-018 is 4 to 8 times more potent than THC [6,7], and adverse outcomes associated with using synthetic cannabinoids containing JWH-018 are more frequent and severe than those arising from cannabis consumption: acute intake of JWH-018 has been shown to cause anxiety, psychosis, hallucinations, and alterations in cognitive abilities [8,9]. Given these potent adverse effects in adults, it is important to understand the short- and long-lasting consequences of JWH-018 exposure during brain development. However, such consequences still remain unknown [10].

Some genes that play important roles in neurodevelopment may modulate the effects of developmental exposure to cannabinoids. Disrupted in schizophrenia 1 (*DISC1*) is a gene in chromosome 1q42.1 that encodes a scaffolding protein with several protein interactions in the brain. Over 100 proteins have been suggested to interact with *DISC1* [11], highlighting the pivotal role of this protein during neurodevelopment [11]. Further, *DISC1* was identified in a Scottish family pedigree, where a translocation between chromosome 1 and 11 (t(1;11)(q42.1;q14.3)) segregated with psychiatric disorders including schizophrenia, depression, and bipolar disorder [12,13].

There is evidence suggesting that alterations due to *DISC1* loss-of-function are exacerbated by exposure to cannabinoids. *Disc1* mutant mice are more susceptible to deficits in fear-associated memory after exposure to THC during adolescence [14]. Perturbation of expression of *Disc1* in astrocytes, but not neurons, increased the effects of adolescent THC exposure on recognition memory assessed in adult mice [15]. Altered expression of *Disc1* and THC exposure caused synergistic activation of the proinflammatory nuclear factor-k-B–cyclooxygenase-2 pathway in astrocytes, leading to secretion of glutamate and dysfunction of GABAergic neurons in the hippocampus [15]. These studies suggest that *Disc1* loss-of-function exacerbates the behavioral effects of THC exposure during adolescence, but no studies have yet examined the effects on earlier developmental exposures, nor the interaction of other cannabinoids (i.e., JWH-018) with *Disc1*.

Here we examine the impact of developmental exposure to JWH018 on anxiety-like behavior using zebrafish as a model. Zebrafish are an ideal animal model to investigate the short- and long-lasting effects of developmental exposure to drugs of abuse: embryos develop externally and are transparent; thus, exposure can be done directly through the water, and effects can be easily monitored. High fecundity of breeding adults provides sample sizes suitable for high-throughput screening experiments with multiple treatments/doses. Although zebrafish cannot develop human psychiatric disorders, they can display behaviors that resemble stress [16], anxiety [17], or drug seeking [18], known as “intermediate phenotypes” or “endophenotypes” [19]. Effects of developmental exposure on larval behavior were assessed using the forced light/dark assay and response to acoustic startle. Adult anxiety-like behavior was assessed using the novel tank diving assay. 

The forced light/dark test is a well-established behavioral assay in zebrafish larvae, where changes in locomotor activity due to alternating bright light/dark depend on the integrity of brain function and the correct development of the visual and nervous system. Transitions from dark to bright light cause an initial rapid locomotor startle response, followed by an abrupt decrease in larval movement (freezing) and subsequent progressive increase in movement that can be interpreted as a measure of recovery to stress-reactivity and anxiety [20]. Transitions from light to dark again induce a startle response, followed by an increase in locomotion and gradual return to baseline. Here, the magnitude of the startle response and the increase in locomotion on transition to light has been interpreted as a measure of anxiety-like responses.

The larval acoustic startle response has been extensively characterized and involves one of two distinct motor behaviors: a short-latency C-bend of the tail, initiating within 5–15 milliseconds of the stimulus, or a slower, long-latency C-bend response, initiating within 20–80 milliseconds. These two motor behaviors use different, possibly overlapping neuronal circuitry [21], but in this study they were measured jointly since a high-speed camera was not available.

When the abrupt sound/vibration stimuli are given repeatedly, zebrafish exhibit iterative reduction in the probability of a startle response, commonly known as habituation. Habituation is the mechanism by which the nervous system filters irrelevant stimuli. It is evolutionarily conserved and present in a wide range of species, from invertebrates such as Aplysia and Drosophila to vertebrates such as rodents [22]. Defective habituation is also associated with neuropsychiatric disorders, such as schizophrenia [23]. In zebrafish assays of response to acoustic startle, a progressive reduction in mean distance travelled with stimulus number gives an indication of habituation [24,25].

Novel tank diving exploits the natural tendency of zebrafish to initially stay at the bottom of a novel tank and gradually move to upper parts of the tank. The degree of “bottom dwelling” has been interpreted as an index of anxiety (greater bottom dwelling meaning greater anxiety), and it is conceptually similar to the rodent open-field and elevated plus maze tasks [17]. Other measures, such as the distance travelled in the tank during the course of the assay and the transitions to bottom of the tank, can give further insights on the hyperresponsiveness to novel environments.

Our two main aims were to interrogate whether the developing central nervous system is susceptible to the effects of JWH-018, and to investigate whether loss-of-function mutations in the *disc1* gene exacerbates the effects of early developmental exposure to JWH-018. We addressed the following research questions: (1) does exposure to JWH-018 during development modulate behavior in larvae zebrafish, (2) are the effects of exposure to JWH-018 during development similar to the effects of THC or nicotine in wild-type larval zebrafish, and (3) are the acute and long-lasting effects of developmental exposure to JWH-018 or THC exacerbated by *disc1* loss of function?

## 2. Materials and Methods

### 2.1. Experimental Design and Timeline

Wild-type zebrafish were exposed to 3 μM JWH-018 (Tocris, Bristol, UK, Cat. No. 1342) from 24 h to 6 days post fertilization (dpf). At 5 dpf (with larvae being exposed to the drug for 96 h), distances travelled during forced light/dark transitions were examined. Importantly, drug was refreshed 3–5 h before behavioral testing and larvae were *in* the drug solution throughout the testing period. At 6 dpf (with larvae being exposed to the drug for 120 h), response and habituation to acoustic startle stimuli were examined. Larvae were also *in* the drug solution during the response and habituation to startle stimuli test, but in this case the drug solution was not refreshed prior to testing since the drug had been refreshed on day five.

To investigate whether the effect of JWH-018 was similar to effects of other psychoactive substances with similar usage profiles and well-characterized effects in zebrafish (namely THC and nicotine), we repeated the experimental protocol and behavioral analysis in wild-type zebrafish larvae using 2 μM THC (Merck, Kenilworth, NJ, USA, Cat. No. T4764) and 0.15 μM nicotine (Sigma, St. Louis, MI, USA, Cat. No. N1019). Drugs were refreshed with the same time course.

**Figure 1:**
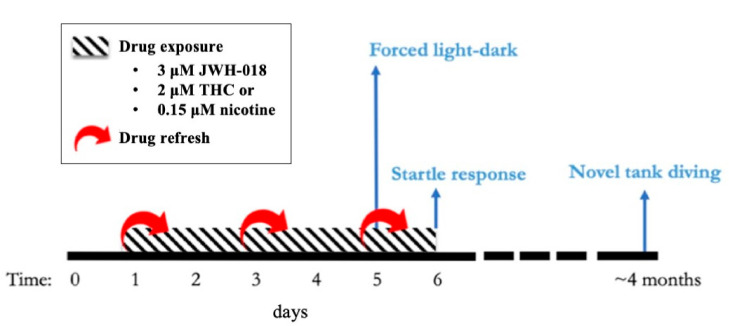
Experimental timeline for developmental exposure to 1-pentyl-3-(1-naphthoyl)indole (JWH-018), delta-9-tetrahydrocannabinol (THC), and nicotine. The behavioral tests performed are represented in light blue.

To examine the potential interactions between JWH-018 and THC exposure and *disc1* mutations in the short and long term, we repeated the developmental exposure to 3 μM JWH-018 and 2 μM THC using *disc1* wild-type and mutant zebrafish, and their behavior was assessed at 5 and 6 dpf (as in experiments with wild-type zebrafish). Furthermore, *disc1* wild-type and mutant zebrafish treated with the cannabinoids but not used for larval behavioral testing were reared to adulthood in normal conditions. At 4 months old, the anxiety-like response of the JWH-018- and THC-exposed vs non-exposed fish was assessed using the novel tank diving procedure. An overview of the study design and experimental timeline is represented in Figure 1.

### 2.2. Animal Maintenance

Zebrafish were housed in a recirculating system (Techniplast, UK) on a 14 h:10 h light/dark cycle (08:30–22:30). The housing and testing rooms were at ∼25–28 °C. Zebrafish were maintained in aquarium-treated water and fed three times daily with live artemia (twice) and flake food (once). Wild-type zebrafish belonged to the Tübingen strain. The *disc1* line (AB background strain) was obtained from the Cecilia Moens lab (Fred Hutchinson Cancer Research Center, Seattle, WA, USA) and was provided by Dr. Jon Wood (University of Sheffield). The mutant allele (*disc1^fh291^*) is caused by a point mutation in exon 2 (T > A) that produces an early stop codon. More information is detailed elsewhere [26].

To breed zebrafish, we placed them in breeding tanks that had either perforated floors or a container with marbles to isolate eggs from progenitors. We moved the animals to breeding tanks in the evening and collected eggs the following morning. Eggs were incubated in Petri dishes at 28 °C with no more than 50 embryos per dish until 5 dpf. Eggs were screened daily to ensure the absence of morphological abnormalities and consistent developmental stage across groups. If reared, larvae were moved to the recirculating system at 6 dpf and fed with commercial fry food. The wild-type and mutant zebrafish larvae raised to adulthood were not the same as the ones used for larval behavioral analysis. The zebrafish raised to adulthood were generated by crossing wild-type and homozygous mutant adults. This enabled us to know the embryos’ genotypes, and to house them separately, removing potential competitive disadvantage of mutants compared to (probably stronger) wild-type zebrafish. More than one tank for each *disc1* genotype group was used to account for possible biases due to tank effects.

All procedures were carried out under license in accordance with the Animals (Scientific Procedures) Act, 1986, and under guidance from the local animal welfare and ethical review board at Queen Mary University of London.

### 2.3. Developmental Drug Exposure

#### 2.3.1. Developmental Exposure to JWH-018, THC, and Nicotine in Wild-type Tübingen Larvae

Since JWH-018 and THC are not soluble in water, JWH-018 was dissolved in DMSO (Sigma-Aldrich, Cat. No. D8418), and THC was provided by the manufacturer in methanol (MeOH). Care was taken to ensure that the final carrier concentration for all samples was 0.1% DMSO (for JWH-018 experiments) and 0.1% MeOH (for THC experiments). To account for potential effects of the carrier substance, we used 0.1% DMSO and 0.1% MeOH, respectively, as control groups. Information about the half-life of THC and JWH-018 in aquatic solutions is scarce; thus, drug and control solutions were changed every 48 h in an attempt to ensure constant drug uptake by the zebrafish embryos and account for potential degradation/oxidation in the water. Zebrafish embryos usually hatch at 48 h post fertilization (hpf); thus, during the first hours of exposure (24 to 48 hpf), drugs had to penetrate the chorion. Previous research suggests that, apart from polymers with very high molecular weight [27], the chorion does not impede the uptake of chemical substances similar to JWH-018. Furthermore, the use of solvents such as DMSO has been reported to facilitate the uptake of chemicals [28].

Drug concentrations for JWH-018 and THC were chosen based on previous studies, where exposure to 2 μM THC led to impaired locomotor response in zebrafish larvae [29], and 3 μM JWH-018 led to behavioral alterations in rodents [30,31] and on pilot dose–response experiments examining effects of acute exposure to JWH-018 or THC on forced light/dark responses at 5 dpf (Supplementary Materials and Figures S3 and S4). Developmental exposure to 0.15 μM nicotine was chosen because previous studies in our lab showed this dose induced increased nicotine preference in adult zebrafish (Supplementary Materials and Figure S5).

#### 2.3.2. Developmental Exposure to JWH-018 in *disc1* Mutant Larvae

Exposure to 3 μM JWH-018 and behavioral testing at 5 and 6 dpf using *disc1* wild-type and mutant zebrafish was carried out as for the wild-type larvae. Larvae were obtained from an in-cross of *disc1* heterozygous zebrafish. Therefore, larvae were a mix of wild-type, homozygous, and heterozygous zebrafish that were randomly allocated in the experimental plates and genotyped after behavioral testing. We performed five independent experiments on five different days. To account for variation across experiments/days, the date of testing was included as a covariate in the analyses.

### 2.4. Behavioral Assays

#### 2.4.1. Forced Light/Dark Test

We conducted forced light/dark tests between 9 a.m. and 4 p.m. with the drug present in the water. We placed larvae in 48-well plates. To reduce stress due to manipulation, we let them acclimatize for at least 1 h in ambient light before testing. Larvae were exposed to alternating light/dark cycles of 10 min: there was an initial 10 min period of dark (baseline), followed by two cycles of 10 min of light and 10 min of dark. This protocol has been used elsewhere [32]. Distances travelled were recorded using Ethovision XT software (Noldus Information Technology, Wageningen, The Netherlands), and data were outputted in one-minute and one-second time bins. Data were fitted to linear mixed models, with total distance travelled as the response variable, experimental variables (e.g., genotype, dose, time) as fixed effects, and fish ID as random effects. Details of the data analysis are given in Supplementary Materials.

#### 2.4.2. Response and Habituation to Startle Stimuli Test

We assessed the response and habituation to startle stimuli between 9 a.m. and 4 p.m. with the drug present in the water (but without drug refresh prior to the test). We used the DanioVision Observation Chamber (Noldus Information Technology, Wageningen, The Netherlands), which contains a dedicated tapping device and set the DanioVision tap stimulus at the highest intensity (intensity level: 8). Larvae were subjected to 10 sound/vibration stimuli over 10 s (1 s interval between each stimulus). For all experiments, distance travelled was recorded using Ethovision XT software (Noldus Information Technology, Wageningen, The Netherlands), and data were outputted in one-second time bins.

As proof of concept, we replicated the experiment by Best and colleagues [24], where 50 stimuli were given using 1, 5, and 20 s inter-stimulus intervals (ISI). Following the habituation paradigm [22], shorter ISI led to faster habituation (Effect of ISI: χ2(2) = 19.04, *p* < 0.0001) (Figure S1).

#### 2.4.3. Novel Tank Diving Test

We transported adult zebrafish (3–4 months) to the behavioral room in their housing tanks and let them acclimatize to the room conditions for at least 1 h before testing. Novel tank diving was assessed as previously described [33]: zebrafish were individually introduced into a 1.5 L trapezoid tank (15.2 cm × 27.9 cm × 22.5 cm × 7.1 cm) (Figure S2) and filmed for 5 min. Their behavior was tracked using EthoVision system (Noldus, Netherlands), and data were outputted in one-minute time bins. Care was taken to ensure that experimental groups were randomized during testing. Behavioral testing was conducted between 9 a.m. and 2 p.m.

We analyzed three behaviors in response to the novel tank: (1) time that zebrafish spent in the bottom third of the tank, (2) total distance that zebrafish travelled in the tank over the 5 min, and (3) number of transitions to the top–bottom area of the tank. Details on the data analysis are in Supplementary Materials.

#### 2.4.4. Code Availability

Code used to analyze the behavioral assays is available at https://github.com/juditperala/Zebrafish-behaviour (accessed on 15 February 2021).

### 2.5. Competitive Allele-Specific PCR (KASP) disc1 Larvae Genotyping

After behavioral testing, DNA was extracted using the hot shock DNA extraction protocol. Since the loss-of-function in *disc1* is caused by a point mutation (see Table 1), we used the competitive allele-specific PCR (KASP) assay (LGC, Biosearch Technologies, Hoddesdon, UK) to genotype the zebrafish.

## 3. Results

No obvious morphological abnormalities or impact on swimming behavior were observed after exposure to THC, JWH-018, or nicotine and prior to behavioral analysis.

### 3.1. Larval Behavior during Developmental Exposure to JWH-018 in Wild-Type Zebrafish

**Figure 2:**
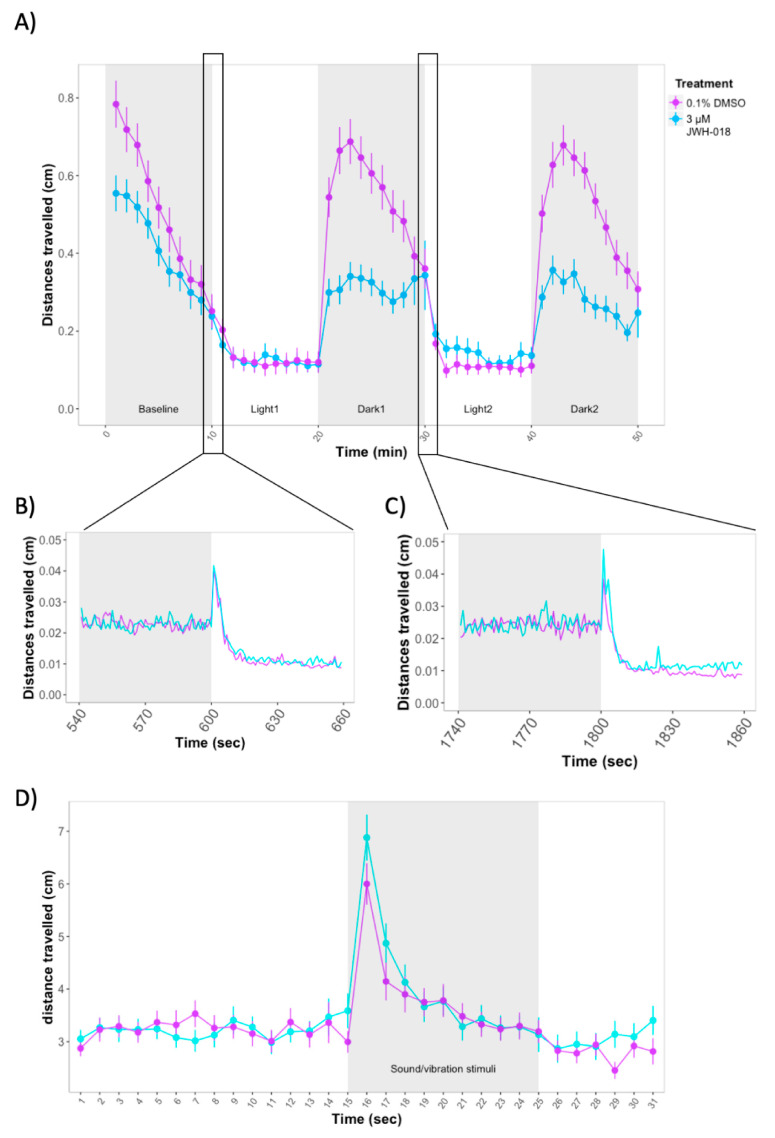
(**A**) Forced light/dark test in 5 days post fertilization (dpf) zebrafish larvae. Sample size: *n* = 64 for each dose group. Each dot represents mean distance travelled per minute. Error bars represent ± SEM. (**B**,**C**) One-second resolution plots of the transitions from dark to light during the forced light/dark test. (**D**) Response and habituation to acoustic startle in 6 dpf zebrafish larvae. Sample sizes: control: *n* = 87, JWH-018-treated: *n* = 81. Each dot represents mean distance travelled per second. Error bars represent ± SEM.

Over the course of the forced light/dark test, time (χ2(1) = 41.27, *p* < 0.0001) and JWH-018 treatment (χ2(1) = 17.53, *p* < 0.0001) predicted distance travelled by 5 dpf larvae (Figure 2A). Exposure to 3 μM JWH-018 impaired locomotion during baseline and dark periods. During the first 10 min of the experiment, treated larvae travelled shorter distances (mean (M) = 0.40, SE = 0.04) than controls (M = 0.50, SE = 0.40) (Effect of treatment during baseline: χ2(1) = 0.04, *p* = 0.04). On transition from light to dark, control larvae sharply increased their locomotion and progressively reduced it, whereas larvae treated with 3 μM JWH-018 did not show as great an increase in movement (M = 0.32, SE = 0.03) as controls (M = 0.55, SE = 0.03) (Effect of treatment during Dark1 and Dark2: χ2(1) = 30.88, *p* < 0.0001). On transition from dark to light period, zebrafish larvae increased the distance travelled during the first seconds after the change in light; however, no differences were observed between JWH-018 treated and non-treated larvae (Figure 2B,C).

We assessed the rate of increase in locomotion during the light periods (measured as the slopes from minute 10 to 20 for the first light period and minute 30 to 40 for the second light period), as well as the decrease in locomotion during the dark periods (measured as the slopes from minute 23 to 30 for the first dark period and minute 43 to 50 for the second dark period). For the two light periods, there were no significant differences between the slopes of treated vs control larvae (*p* > 0.05). However, for the dark periods, the slopes were significantly steeper in control vs treated larvae (Dark1: *F*(1) = 7.959, *p* = 0.006. Dark2: *F*(1) = 7.966, *p* = 0.006).

We next assessed the response to repeated startle stimuli at 6 dpf. There were no significant differences between 3 µM JWH-018-treated and control larvae in distance travelled before and during the stimuli (*p* > 0.05) (Figure 2D).

### 3.2. Larval Behavior during Developmental Exposure to THC and Nicotine in Wild-Type Zebrafish

**Figure 3:**
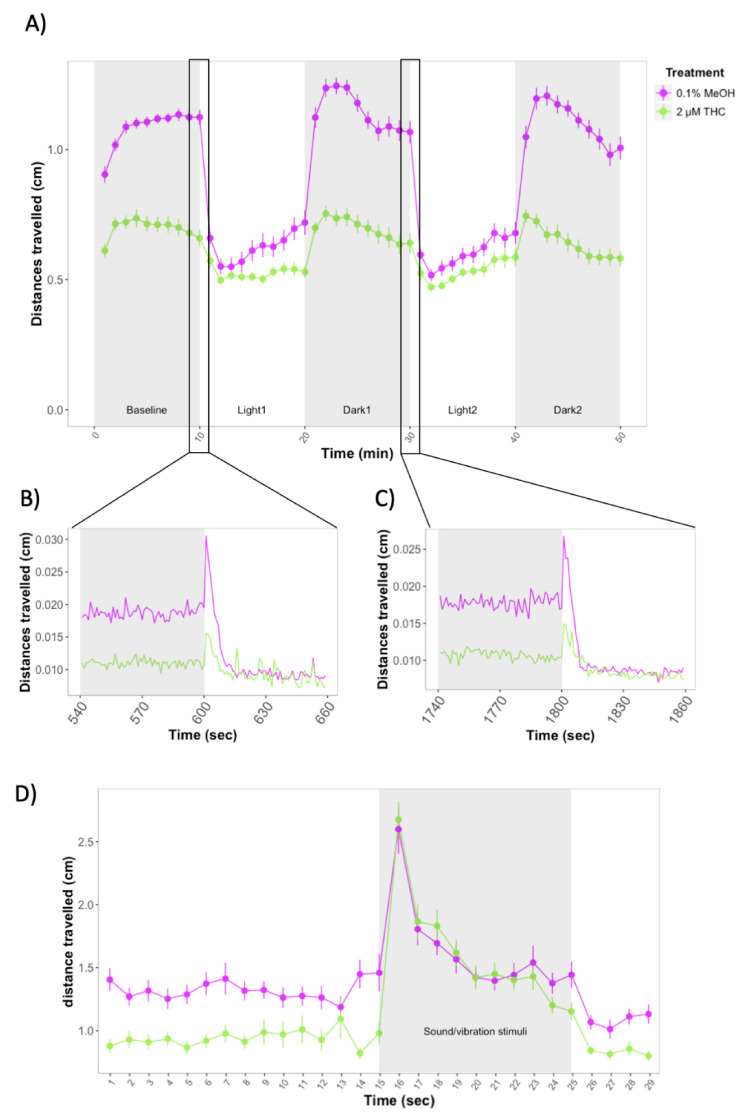
(**A**) Forced light/dark test in wild-type zebrafish exposed to 2 μM THC. Sample size: *n* = 48 for each dose group. Each dot represents the mean of the total distance travelled per minute. Error bars represent ± SEM. (**B**,**C**) One-second resolution plots of the transitions from dark to light during the forced light/dark test. (**D**) Distances travelled by control and THC-treated larvae before and after exposure to 10 sound/vibration stimuli. Figure shows mean distances travelled in one-second time bins. Each dot represents mean distance travelled per second.Error bars represent ± SEM. Sample sizes: *n* = 48 per dose group.

We investigated whether the behavioral effects of exposure to nicotine or THC during development were similar to those of JWH-018. Exposure to 2 μM THC led to impaired locomotion of larvae, similar to the effects observed for the JWH-018 treatment. Distances travelled over the course of the experiment were much shorter for THC-treated larvae (M = 0.62, SE = 0.02) compared to controls (M = 0.91, SE = 0.02) (Effect of THC treatment: χ2(1) = 120.89, *p* < 0.0001). The differences between treated vs control larvae were consistent for baseline, light, and dark periods (Figure 3A). During the first seconds of the transitions from dark to light, zebrafish increased the distances travelled, but the increase was significantly lower in THC-treated zebrafish compared to non-treated animals (Figure 3B,C).

Treatment with 2 μM THC also affected larval recovery slopes during the first light period. Slopes for control larvae were steeper (M = 0.02, SE = 0.006) than for THC-treated larvae (M = 0.004, SE = 0.006) (*F*(1) = 5.397, *p* = 0.0223). However, there were no significant differences between slopes of treated vs control larvae for the second light period or for the two dark periods (*p* > 0.05).

Zebrafish larvae treated with 2 μM THC were less active during the first 30 s of the repeated startle response assay, before any stimulus was given (Effect of THC treatment: χ2(1) = 15.31, *p* < 0.0001). However, during the ten sound/vibration stimuli, larvae had similar locomotor activity (*p* > 0.05) (Figure 3D).

**Figure 4:**
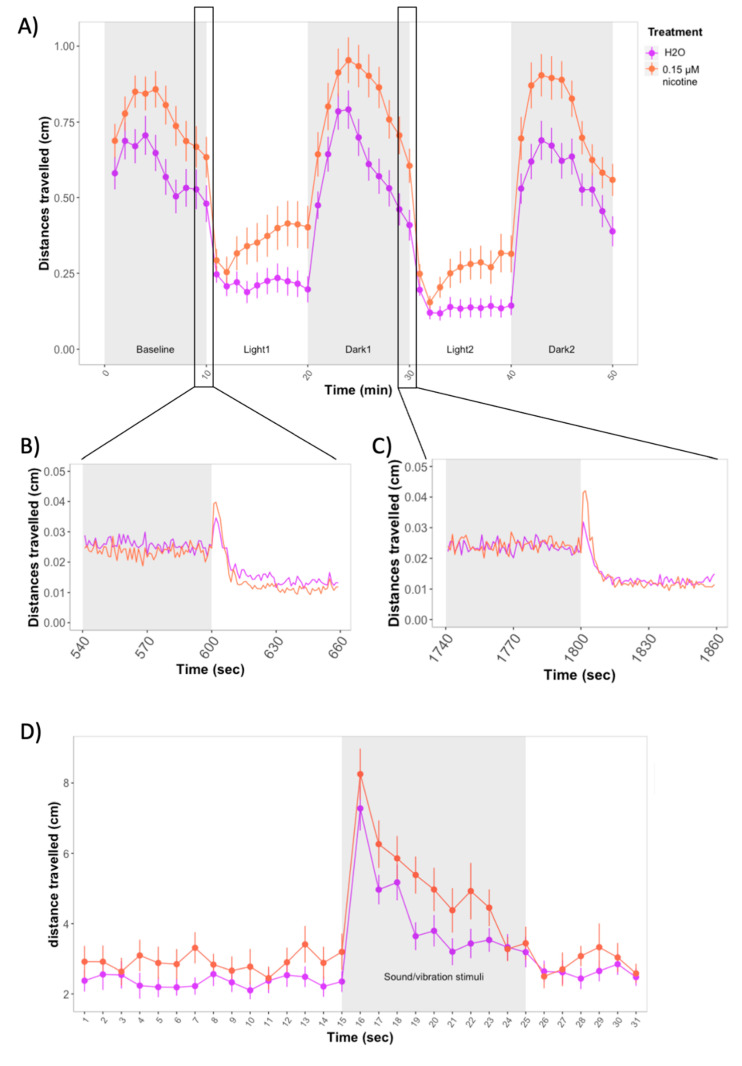
(**A**) Forced light/dark test in wild-type zebrafish exposed to 0.15 μM nicotine. Sample size: *n* = 48 for each dose group. Each dot represents the mean of the total distance travelled per minute. Error bars represent ± SEM. (**B**,**C**) One-second resolution plots of the transitions from dark to light during the forced light/dark test. (**D**) Distances travelled by control and nicotine-treated larvae before and after exposure to 10 sound/vibration stimuli. Figure shows mean distances travelled in one-second time bins. Each dot represents mean distance travelled per second. Error bars represent ± SEM. Control: *n* = 23, treated with 0.15 μM nicotine: *n* = 23.

In contrast to JWH-018 and THC, exposure to 0.15 μM nicotine produced an increase in distances travelled over the course of the forced light/dark assay (χ2 (1) = 16.04, *p* < 0.0001). The increased distances travelled by nicotine-treated larvae were significant for baseline, dark, and light periods (*p* < 0.0001) (Figure 4A).

There was a qualitative difference between control and treated zebrafish in the slopes during light periods, as nicotine-treated zebrafish seemed to recover more quickly. However, the difference in slope between nicotine-treated and control zebrafish was not significant during light nor dark periods (*p* > 0.05). During the first seconds of the transitions from dark to light, zebrafish increased the distances travelled; the increase was higher among larvae treated with nicotine compared to non-treated larvae, but differences did not reach significance (Figure 4B,C).

Similar to the response seen during the forced light/dark test, zebrafish treated with 0.15 μM nicotine showed increased locomotion during the acoustic startle assay. The effect was significant during stimuli (χ2(1) = 4.00, *p* = 0.04), but not during the first 15 s before the stimuli (*p* > 0.05) (Figure 4D).

### 3.3. Larval Behavior during Developmental Exposure to JWH-018 in Wild-Type and Mutant disc1 Zebrafish

**Figure 5:**
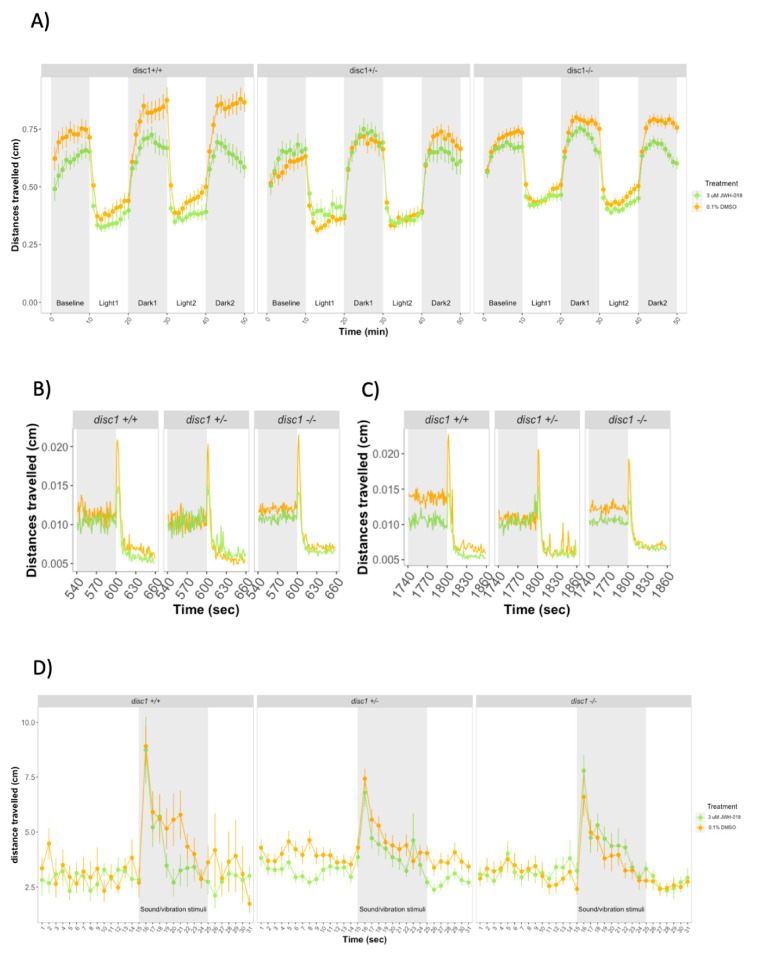
(**A**) Forced light/dark test in 5 dpf wild-type and *disc1* loss-function mutant larvae. Sample sizes for each group: control *disc1* +/+: *n* = 30, JWH-018 *disc1* +/+: *n* = 34, control *disc1* +/–: *n* = 33, JWH-018 *disc1* +/–: *n* = 27, control *disc1* –/–: *n* = 107, JWH-018 *disc1* –/–: *n* = 92. Each dot represents mean distance travelled per minute. Error bars represent ± SEM. (**B**,**C**) One-second resolution plots of the transitions from dark to light during the forced light/dark test. Each dot represents mean distance travelled per second. (**D**) Response and habituation to startle stimuli test in 6 dpf control and JWH-018-treated wild-type and *disc1* mutant larvae. Sample sizes: control *disc1* +/+: *n* = 15, JWH-018 *disc1* +/+: *n* = 13, control *disc1* +/–: *n* = 47, JWH-018 *disc1* +/–: *n* = 47, control *disc1* –/–: *n* = 22, JWH-018 *disc1* –/–: *n* = 22.

Over the 50 min of the forced light/dark test, JWH-018 treatment (χ2(1) = 12.51, *p* < 0.0001) and time (χ2(1) = 72.83, *p* < 0.0001) were significant predictors of distance travelled. Although *disc1* wild-type larvae travelled greater distances than mutants, genotype effects were not significant (χ2(1) = 4.9, *p* = 0.08) (Figure 5A).

During baseline, neither treatment nor genotype affected distances travelled (*p* > 0.05). During the dark periods, wild-type and *disc1* homozygous (but not *disc1* heterozygous larvae) travelled shorter distances when exposed to JWH-018 (Effect of JWH-018 treatment: χ2(1) = 16.17, *p* < 0.0001). The effect of JWH-018 was also observed during the transitions from dark to light periods, where treated wild-type and *disc1* mutant larvae travelled shorter distances compared to controls. However, there was no difference in the increase above baseline across treatment or genotypes (Figure 5B,C). Mutations in *disc1* affected the slope during the first dark period (Effect of genotype: χ2(2) = 4.22, *p* < 0.0156) but not during the second one.

During light periods, there was a main effect of JWH-018 treatment (χ2(1) = 4.57, *p* = 0.032): larvae exposed to JWH-018 travelled shorter distances than control larvae. However, there were no significant main effects of *disc1* genotype nor significant interactions between genotype and JWH-018 on distances travelled nor on the slopes calculated during light periods.

After 24 h from the last JWH-018 drug refresh, treated and control larvae showed no significant differences in distances travelled before or during the startle stimuli. There were no significant differences across *disc1* genotype groups (Figure 5D).

### 3.4. Larval Behavior during Developmental Exposure to THC in Wild-Type and Mutant disc1 Zebrafish

**Figure 6:**
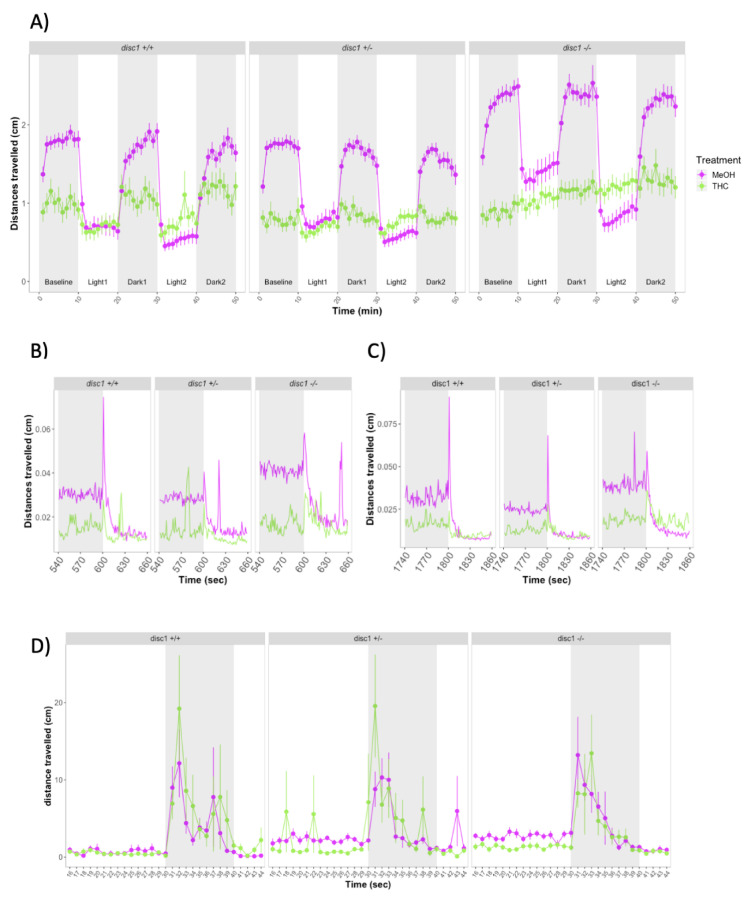
(**A**) Forced light/dark test in 5 dpf wild-type and *disc1* loss-function mutant larvae. Sample sizes for each group: control *disc1* +/+: *n* = 39, THC *disc1* +/+: *n* = 29, control *disc1* +/–: *n* = 41, THC *disc1* +/–: *n* = 45, control *disc1* –/–: *n* = 40, THC *disc1* –/–: *n* = 29. Each dot represents mean distance travelled per minute. Error bars represent ± SEM. (**B**,**C**) One-second resolution plots of the transitions from dark to light during the forced light/dark test. Each dot represents mean distance travelled per second. (**D**) Response and habituation to startle stimuli test in 6 dpf control and THC-treated wild-type and *disc1* mutant larvae. Sample sizes: control *disc1* +/+: *n* = 36, THC *disc1 +/+: n* = 35, control *disc1* +/–: *n* = 36, THC *disc1* +/–: *n* = 35, control disc1 –/–: *n* = 36, THC *disc1* –/–: *n* = 36.

Over the 50 min of the forced light/dark test, THC treatment (χ2(1) = 50.75, *p* = 1.05 × 10^–12^) and *disc1* genotype (χ2(1) = 45.16, *p* = 1.56 × 10^–10^) were significant predictors of distance travelled (Figure 6A). THC produced a strong reduction in distances travelled across the whole experiment. The effects of THC were observed both at both one-minute and one-second time bin resolution (Figure 6B,C). The effects of *disc1* genotype were dependent on the THC treatment: in drug free conditions, *disc1* homozygous larvae showed increased locomotion compared to wild-type and heterozygous larvae. However, in the presence of THC, wild-type and heterozygous larvae followed the usual light/dark locomotion pattern, whereas *disc1* homozygous larvae showed a steady increase in locomotion.

During light periods, THC treatment and *disc1* genotype did not affect the slopes. For all genotypes in the absence of THC, distances travelled in the second light period was significantly less than in the first light period (Effect of light period number: χ2(1) = 62.76, *p* = 2.34 × 10^–15^). The reduction in locomotion during the light period was greater in homozygous *disc1* fish and was observed only for non-treated larvae (Effect of three-way interaction between light period, disc1 genotype and THC treatment: χ2(2) = 61,10, *p* = 5.40 × 10^–14^). We observed a significant effect of genotype during the first dark periods (χ2(1) = 3.92, *p* = 0.02) and interaction of genotype and treatment during both dark periods (Dark period 1: χ2(1) = 3.51, *p* = 0.03; Dark period 2: χ2(1) = 7.22, *p* = 0.0009).

There was a main effect of treatment (χ2(1) = 63.93, *p* < 0.0001) and genotype (χ2(1) = 32.06, *p* < 0.0001) on locomotion during the first 30 sec of the repeated startle response assay, before any stimulus was given, and a Treatment x Genotype interaction (χ2(1) = 15.67, *p* < 0.0004). Wild-type larvae treated with THC did not differ in their basal movement compared to untreated larvae. However, 2 μM THC exposure significantly reduced locomotion in *disc1* heterozygous and homozygous mutant larvae. During the ten sound/vibration stimuli, larvae had similar locomotor activity (*p* > 0.05) (Figure 6D).

### 3.5. Adult Behavior after Developmental Exposure to JWH-018 in Wild-Type and Mutant disc1 Zebrafish

**Figure 7:**
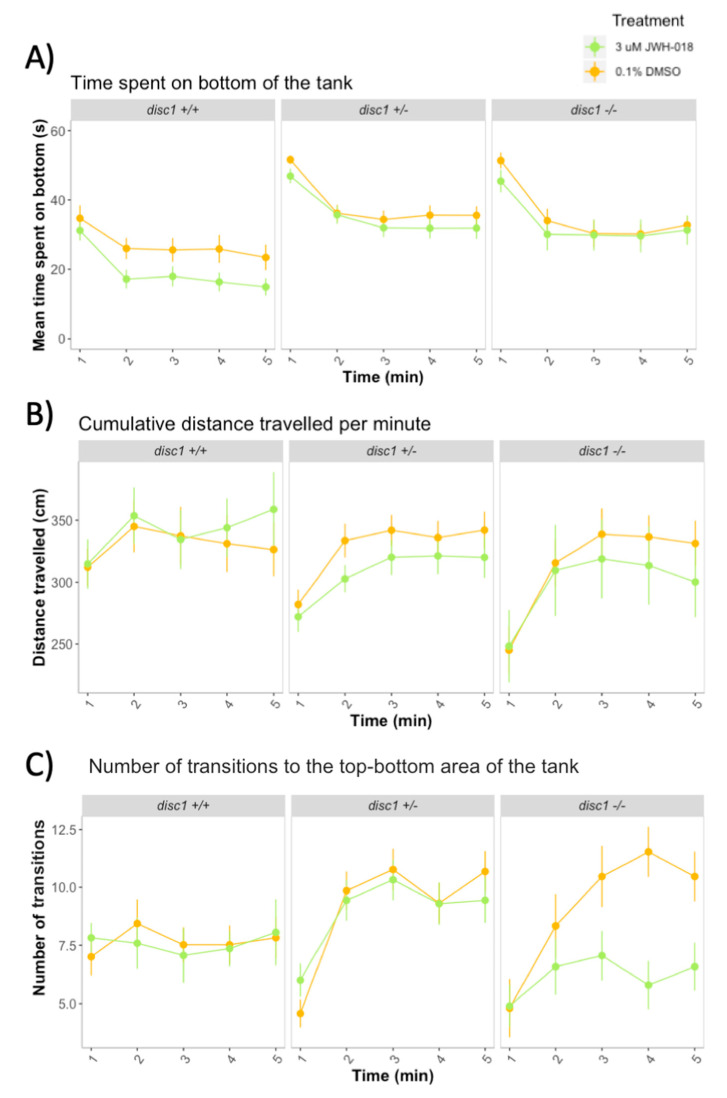
Novel tank diving response in adult wild-type and mutant *disc1* zebrafish after developmental exposure to 3 μM JWH-018. (**A**) Time spent on the bottom of the tank, (**B**) Cumulative distance travelled (**C**) Number of transitions between the top and bottom of the tank. Sample sizes for each group: control *disc1* +/+: *n* = 23, JWH-018 *disc1* +/+: *n* = 17, control *disc1* +/–: *n* = 35, JWH-018 *disc1* +/–: *n* = 34, control *disc1* –/–: *n* = 15, JWH-018 *disc1* –/–: *n* = 19. Error bars represent ± SEM.

The *disc1* genotype affected the behavioral response during the novel tank assay (Figure 7). Wild-type zebrafish spent less time on the bottom of the tank than homozygous and heterozygous *disc1* mutants (Effect of genotype: χ2(14) = 119.40, *p* < 0.0001) (Figure 7A). Distances travelled over the five minutes of the experiment were also different across *disc1* genotypes (Figure 7B): while wild-type zebrafish did not differ in the distance travelled over time, zebrafish heterozygous and homozygous for *disc1* moved less during the first minute and increased later the distance travelled (Effect of genotype by time interaction: χ2(14) = 18.15, *p* = 0.02). The number of transitions between the bottom and top area of the tank over the five minutes of the experiment remained similar for wild types but increased for heterozygous and homozygous zebrafish (Effect of genotype by time interaction: χ2 (8) = 22.93, *p* < 0.0001) (Figure 7C).

Developmental exposure to JWH-018 reduced the time spent on the bottom of the tank (Effect of JWH-018 treatment: χ2(1) = 11.31, *p* < 0.0001). The effect was stronger for wild-type than for mutant zebrafish (Figure 7A), but there were no significant genotype by JWH-018 treatment interactions (*p* > 0.05). Developmental exposure to JWH-018 did not affect the distance travelled nor the number of transitions between the top and bottom area of the tank for wild-type and heterozygous *disc1* zebrafish (Figure 7B,C) (*p* > 0.05). For homozygous *disc1* zebrafish, treatment with JWH-018 decreased the number of top–bottom transitions but the interaction between genotype and JWH-018 treatment was not significant (Figure 7C).

In contrast to animals exposed to 3μM JWH-018, which spent less time on the bottom of the tank, developmental exposure to 2μM THC and 0.15μM nicotine had no significant effect on tank diving response in wild-type fish (Figure S6).

## 4. Discussion

This study used zebrafish as an animal model to investigate the behavioral effects of developmental exposure to JWH-018, the main psychoactive compound of synthetic cannabinoids, on larval and adult behavior in wild-type and *disc1* mutant fish. Wild-type larvae exposed to JWH-18 or the related cannabinoid THC showed reduced locomotion during behavioral testing but—with the possible exception of a reduced rate of recovery on light to dark transition in a forced light/dark test in the presence of JWH-018—their anxiety-like behavior and response to repeated sound/vibration stimuli were not altered. No locomotor effects were observed in adult wild-type fish that had been developmentally exposed to either cannabinoid, but fish exposed to JWH-018 exhibited decreased anxiety-like behavior in the novel tank diving assay, suggesting that developmental exposure to JWH-018 can have lasting effects on behavior. At larval stages, loss-of-function in *disc1* increased sensitivity to effects of THC but not of JWH-018. During adulthood, loss-of-function in *disc1* increased zebrafish anxiety-like responses but did not moderate sensitivity to the effects of JWH-018 (See Table 2 for summary).

Stimulatory and depressant responses elicited by neuroactive drugs used by humans can be modeled in zebrafish larvae. For example, exposure to adrenaline—a neuro-stimulant—increased the locomotor activity in the forced light/dark test, whereas tricaine—a CNS depressant—decreased it [34]. In this study, we show developmental exposure to JWH-018 reduced the locomotor activity of 5 dpf wild-type zebrafish during dark periods in the forced light/dark test. The effects of JWH-018 were similar to the effects of THC but opposite to the effects of nicotine. The results for THC and nicotine are in line with previous studies showing a reduction in locomotion after exposure to THC [29] and an increase in locomotion after exposure to nicotine [35]. We hypothesize that cannabinoids may produce a CNS depressant effect, whereas exposure to nicotine has a behaviorally stimulant effect in zebrafish larvae. However, we cannot rule out that these drugs affected zebrafish behavior via impairment/activation of motor neurons or toxicity effects [36].

Alterations in the typical response of larvae to transitions between light and dark periods can be used to study the anxiety-like response in zebrafish. Others have interpreted the distance travelled in the dark period immediately following light exposure as a measure of anxiety or the distance travelled during the startle response on dark to light transition—the greater the distance moved, the more anxious [37]. Here we found that larvae treated with JWH-018 moved significantly less than controls, suggesting possible anxiolytic effects. However, since this interpretation is problematic when there are clear effects on locomotion, we examined the slopes during both light and dark periods, which represent how quickly zebrafish larvae recover from a startle stimulus (i.e., light to dark transitions, or dark to light transitions). We would argue that this latter approach, particularly when applied to the light period, provides a measure of stress and anxiety less biased by locomotor effects, such that a more rapid rate of recovery reflects reduced anxiety. Using this interpretation, our results show JWH-018-exposed larvae recovered more slowly during the first dark period, and THC-exposed larvae recovered more slowly during the first light period—suggesting an anxiogenic effect of both cannabinoids, and larvae exposed to nicotine tended to (non-significantly) recover more quickly in both the light and dark—suggesting an anxiolytic effect of nicotine. Previous studies of the impact of acute exposure to THC or nicotine on the novel tank diving response in adult zebrafish support our interpretation of the forced light/dark data: compared to controls, fish pre-exposed to THC spent more time on the bottom of the tank, consistent with an anxiogenic effect [38], whereas animals pre-exposed to nicotine spent less time on the bottom of the tank, consistent with an anxiolytic effect [39]. However, as both JWH-018 and THC had marked effects on locomotion during the forced light/dark assay, caution should be taken when drawing conclusions from these data.

Using distance travelled in response to acoustic startle as an indication of anxiety-level, no effect of developmental exposure to THC or JWH-018 was observed, nor was there any effect on baseline locomotion in wild-type Tübingen larvae. As acoustic startle was conducted 24 h after the last drug refresh, these latter data suggest any effects on locomotion or anxiety during the forced light/dark assay are due to the presence of the drug rather than adaptive changes.

When anxiety-like behavior was assessed during adulthood, we observed wild-type zebrafish developmentally exposed to JWH-018 spent less time on the bottom of the tank, suggesting they were less anxious when placed in a new environment compared to nonexposed animals. These results differ from previous reports, suggesting anxiogenic effects due to drug withdrawal in zebrafish [40,41]. However, the time between drug exposure and analysis of behavioral effects in these withdrawal studies is very different to the current study, limiting their comparability. Further, none of the previous studies exposed fish to JWH-018, nor did they expose them at early developmental stages and test months after withdrawal. In our study, exposure to JWH-018 started at 24 h post fertilization, a period in which the main zebrafish brain structures (i.e., forebrain, midbrain, and hindbrain) are formed, but finer structures are still to be defined [42]. It is possible that exposures at such early ages lead to changes in gene expression and neurotransmission that impact on neurodevelopment and differ from the adaptive mechanisms occurring during other developmental periods—such as adolescence. Our results showing lack of impact of developmental exposure to nicotine on anxiety-like behavior is also at odds with findings in rodents [43] and our previous findings that developmental exposure to 0.15 μM nicotine from 1 to 7 days led to increased sensitivity to the rewarding effects of nicotine. The reason for this difference is yet to be established, but it may reflect the relatively low concentration used and limited duration of exposure.

Although no effect of developmental exposure to nicotine or THC at the concentrations used here were observed on tank diving responses in wild-type fish, despite noticeable impact on locomotor response during exposure, the direction of the nicotine effects is in line with previous research. Our results show adult zebrafish developmentally exposed to nicotine spent more time on the bottom of the tank during the first minute of the assay, consistent with previous studies showing an increase in adult fish startle response when embryos were exposed to nicotine [44].

The novel tank diving results show that adult, but not larval, zebrafish with loss-of-function mutations in *disc1* showed increased anxiety-like responses compared to wild types. These results are in line with another study showing abnormal adult stress response in this mutant line [45] and support the role of *disc1* in zebrafish hypothalamus-pituitary-interrenal axis function [45]. Previous research in zebrafish have shown that alterations in *disc1* causes alterations in the specification of oligodendrocytes and neurons [46] and in the migration and differentiation of the neural crest (the cells that form the craniofacial cartilage and connective tissue of the head) [47]. Alterations in these processes could also underlie the alterations in behavior we observed. DISC1 is a scaffolding protein that interacts with many other proteins and regulates the formation, maintenance, and correct regulation of neural networks [11]. Given the number of interacting proteins, the specific biological mechanisms by which DISC1 acts is a complex question out of the scope of this study. However, our work supports zebrafish as a model in which to investigate the role of DISC1 in stress and neurodevelopment.

We showed no evidence of *disc1* altering sensitivity to the effects of JWH-018 at larval or adult stages; the effects of JWH-018 were less appreciable in mutant zebrafish but did not reach statistical significance. However, *disc1* genotype affected larval responses to forced light/dark transition in the presence of THC and baseline locomotion in the acoustic startle assay one day after withdrawal from THC. In both cases, THC was found to have greater effects in *disc1* mutants than wild-type siblings. These findings are consistent with studies in mice reporting synergistic effects between THC and alterations in *Disc1*. The explanation for the difference in genotype effects on response to JWH-018 compared to THC exposure is not clear. However, disparities in the psychoactive compound (JWH-018 vs THC; full vs partial agonist) and kinetics may underlie these differences. Further work using different species is needed to replicate our findings. One limitation of our study that may be of relevance in this regard is that we did not explore possible sex differences. In both human and rodent studies, females have been shown to be more sensitive to the behavioral and physiological effects of cannabinoids [48]. In addition, dominant-negative *disc1* mutant mice show sex-dependent differences in social behavior and expression of cannabinoid receptors [49].

It is notable that the slopes in the untreated fish in the forced light/dark experiments differed between treatments; for example, the slope in the light period was much flatter in the JWH-018 and nicotine experiments than the THC experiments. As treatments were done in parallel balanced across plates, this difference likely reflects differences in carrier (DMSO vs methanol vs water) [50]. Interestingly, the behavioral pattern of the Tübingen wild types and the *disc1* wild-type larvae in the forced light/dark test also differed in that untreated Tübingen larvae tended to show a decline in locomotion during the dark periods, whereas *disc1* wild-type larvae maintained high levels of locomotion or even showed an increase over the dark period. Since they belonged to different zebrafish strains (Tübingen vs AB), differences may be due to their genetic background. It is also possible that factors such as time of day or time of year contributed to observed differences between Tübingen and *disc1* strains, as the experiments were conducted separately. Although care was taken to ensure that time of drug exposure prior to testing, time of behavioral testing, and developmental stages were similar across experiments, these experimental parameters are known to affect zebrafish behavior [51].

JWH-018 did not affect the behavioral response of zebrafish larvae at 6 dpf. To maintain a gap of 48 h between each refresh, we did not refresh the drug before testing at this age, and therefore the absence of behavioral phenotype could be due to (1) JWH-018 being metabolized very quickly and no accumulation in the larvae, so after 24 h there was no noticeable effect and/or (2) JWH-018 is not stable in water and its psychotropic properties were lost after a few hours in the water. As our data suggest acute effects of JWH 018 are lost after 24 h in the water, it is possible that an altered exposure paradigm would impact behavioral outcomes. However, it is difficult to predict whether a lesser or greater effect on adult behavior would be seen: some studies for other centrally active drugs suggest an intermittent exposure regime increases adaptation [52,53]. In order to disentangle these scenarios, liquid chromatography–mass spectrometry analyses could be used to measure the concentrations of the drug in the water and in zebrafish tissue. It is also possible that the repeated administration of JWH-018 produced tolerance to behavioral effects in zebrafish larvae, since it has been shown in rodents that repeated injection of similar doses of JWH-018 produced tolerance to its hypothermic and cataleptic effects [31]. Future studies where the behavioral effect of repeated vs single exposures is compared would be valuable to examine the tolerance of different drugs.

## 5. Conclusions

This is the first study looking at the behavioral effects of early developmental exposure to JWH-018 and the interaction with loss-of-function mutations in *disc1*. Our results suggest that exposure to drugs of abuse during early-development leads to long-term behavioral changes in zebrafish. However, further studies in human populations and other models are needed to confirm these findings. Our results align with previous research suggesting that functional abnormalities in DISC1 have a behavioral impact and report no evidence of synergistic effect between developmental exposure to JWH-018 and *disc1*. These results pave the way to study molecular mechanisms by which *disc1* and developmental exposure to JWH-018 act and give little evidence for interaction between *disc1* and developmental exposure to synthetic cannabinoids.

## Figures and Tables

**Table 1:** Genomic sequence surrounding the point loss-of-function mutation (T > A, in red) for *disc1*.

Position	Genomic Sequence Surrounding the SNP Polymorphism
13:49125537- 49125647	AGAGGGTTTCGAGAGAGACAACTCATCAAAGTC TTCAAATAAACACCATT[T/A]GCATGATGAGGAG GACAATTTACCAGTGCAATCACGTGATGTTTTCAATT

**Table 2:** Summary of results. (− −) (++) indicate a decreased or increased response in the presence of drug, respectively.

Behavioral Assay	JWH-018	THC	*disc1*	*disc1* × JWH-018 Interaction	*disc1* × THC Interaction
Forced Light/Dark assay at 5 dpf					
Basal locomotion	(− −)	(− −)	No effect	No	Yes
Light period locomotion	No change	(− −)	No effect	No	Yes
Light period slope	No change	(−)	No effect	No	No
Dark period locomotion	(− −)	(– –)	No effect	No	Yes
Dark period slope	(− −)	No change	(−)	No	Yes
Acoustic startle at 6 dpf					
Basal locomotion	No change	(− −)	No effect	No	Yes
Across taps	No change	No change	No effect	No	No
Adult tank diving—time on bottom	(− −)	No change	(+ +)	No	No

## Data Availability

All zebrafish data generated or analyzed during this study are included in the manuscript and supporting files. Source data files for all figures can be obtained from c.h.brennan@qmul.ac.uk or judit@perala.es.

## Supplementary Materials

The following are available online at https://www.mdpi.com/2218-273X/11/2/319/s1, Figure S1: Response and habituation to startle stimuli test with different interstimulus intervals (ISI) in wild-type zebrafish larvae, Figure S2: Tank used for novel tank diving assay. Figure S3. Forced Light/Dark test in 5 dpf wild-type zebrafish larvae after acute exposure to JWH-018 (Drug exposure: 3–4 h prior experiment). Sample sizes *n* = 40 per experimental group. Error bars represent standard error of the mean. Figure S4. Forced Light/Dark test in 5 dpf wildtype zebrafish larvae after acute exposure to THC (Drug exposure: 3–4 h prior experiment). Sample sizes *n* = 48 per experimental group. Error bars represent standard error of the mean. Figure S5. 5 μM nicotine-induced place preference in adult zebrafish is exacerbated by developmental exposure to 0.15 μM nicotine (from 2–7 dpf). Sample size *n* = 17 to 20 fish per experimental group. Figure S6. Novel tank diving response in adult wild-type zebrafish after developmental exposure to 2μM THC and 0.15 μM nicotine. Sample sizes for each group: control THC: *n* = 30, developmentally exposed to THC: *n* = 40, control nicotine: *n* = 79, developmentally exposed to nicotine: *n* = 53. Error bars represent ± SEM.
