# Supplementary material for: Behavioral Effects of Developmental Exposure to JWH-018 in Wild-Type and Disrupted in Schizophrenia 1 (disc1) Mutant Zebrafish
Source: Biomolecules. 2021 Feb 19;11(2):319. doi: 10.3390/biom11020319 (PMC7922669; doi:10.3390/biom11020319)
Supplement: Supplementary file 1 [file biomolecules-11-00319-s001.pdf]

biomolecules

MDPI

SUPPLEMENTARY MATERIAL AND APPENDICES A-B

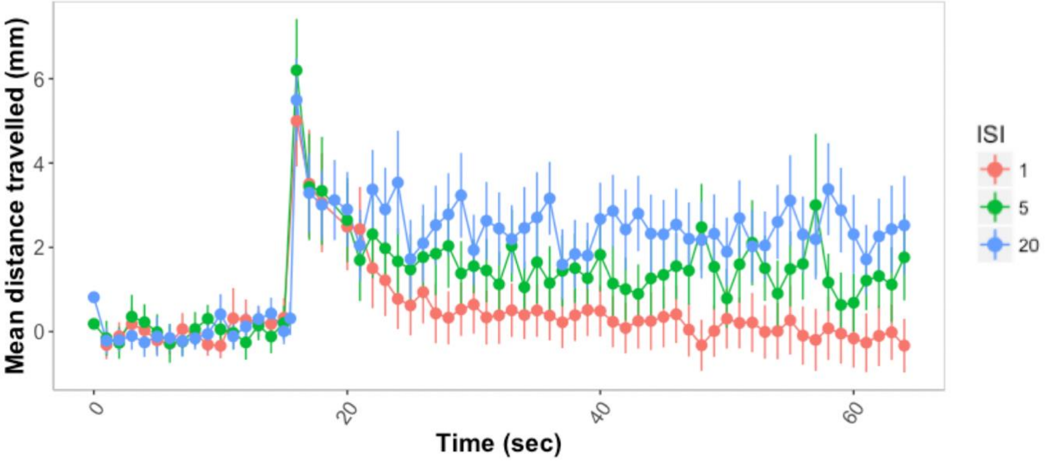

Figure S1. Response and habituation to startle stimuli test with different interstimulus intervals (ISI) in wild type zebrafish larvae. The first stimulus is given at second 15.

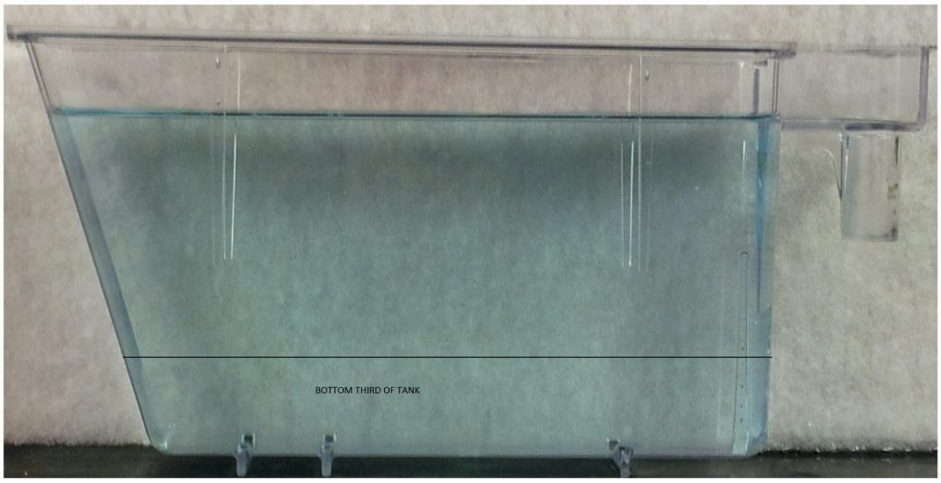

Figure S2. Tank used for novel tank diving assay.

Appendix A:

Effects of acute exposure to three different concentrations of JWH-018 on larval behavior.

Over the course of the experiment, time [$\chi 2(1)=32.59$, $p<0.0001$] and JWH-018 dose [$\chi 2(2)=57.92$, $p<0.0001$] were significantly associated with the distance travelled by larvae (Figure S3).

Treatment with 3 μM JWH-018 impaired larvae locomotion, which was especially noticeable during dark periods [$\chi 2(2)=86.65$, $p<0.0001$].

During baseline, 3 μM JWH-018 treated larvae moved significantly less (M=0.26, SE=0.05) than controls (M=0.79, SE=0.05), suggesting that the effects of the drug were independent of exposure to stressful stimuli (such as light). There were no differences between DMSO and 0.03 μM JWH-018 treated larvae during dark1 and dark2.

During light, the distances travelled plunged, reducing the differences across treatment groups. Results for the linear mixed model reported no main effect of dose ($p\geq 0.83$), but a significant dose by time interaction [$\chi 2(2)=73.55$, $p<0.0001$]. The interaction effect was caused by the impairment in recovery due to JWH-018 exposure: Whereas control fish steadily increased the distances travelled during ligh, larvae treated with 3 μM JWH-018 were unable to show signs of recovery. Slopes for larvae treated with 0.03 μM JWH-018 were lower (Light1: M=0.02, SE=0.011, Light2: M=0.04, SE=0.014) than those for control larvae (Light1: M=0.053, SE=0.011, Light2: M=0.081, SE=0.014), suggesting impairment in recovery, however differences were not statistically significant ($p\geq 0.05$).

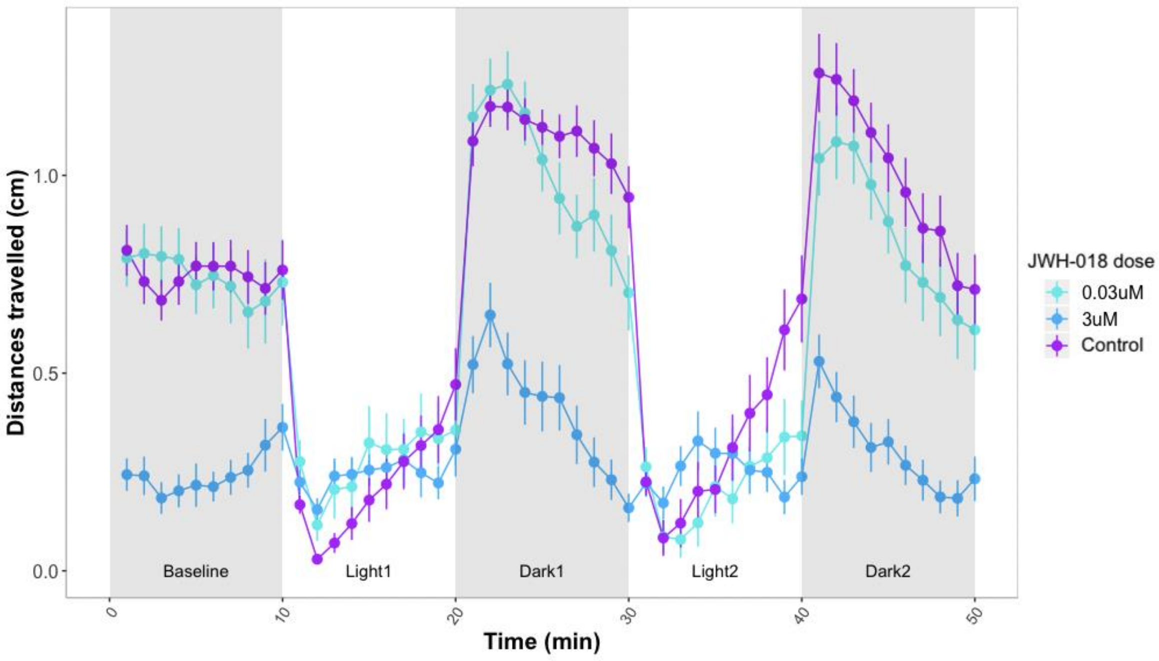

Figure S3. Forced Light-Dark test in 5dpf wildtype zebrafish larvae after acute exposure to JWH-018 (Drug exposure: 3-4 hours prior experiment). Sample sizes n=40 per experimental group. Error bars represent standard error of the mean.

Effects of acute exposure to three different concentrations of THC on larval behavior.

Analysis of the 50 minutes experiment showed that time [$\chi2(1)=99.91$, $p<0.0001$] and dose [$\chi2(2)=19.13$, $p<0.0001$] had a significant effect on distance travelled (Figure S4).

Five dpf larvae treated with 2 μM THC travelled shorter distances over the course of the experiment. This difference was more pronounced during dark periods, where fish treated with 2 μM THC were not able to increase their locomotor activity to the same magnitude as control or 0.05 μM THC treated larvae [$\chi2(2)=14.241$, $p=0.0008$].

During light, all the larvae reduced their locomotion to ~0.5 cm average distance travelled during one minute. Treatment with THC affected the 'recovery' of larvae during the light phase. For the first light period there were no significant differences in the slopes across THC dose groups. However, during the second light period, slopes for fish treated with 2 μM THC were significant lower (M=0.009, SE=0.007) than for fish treated with 0.05 μM THC (M=0.01, SE=0.007) or 0.1% MeOH Control (M=0.04, SE=0.007) [$F(2)=4.418$, $p=0.0138$].

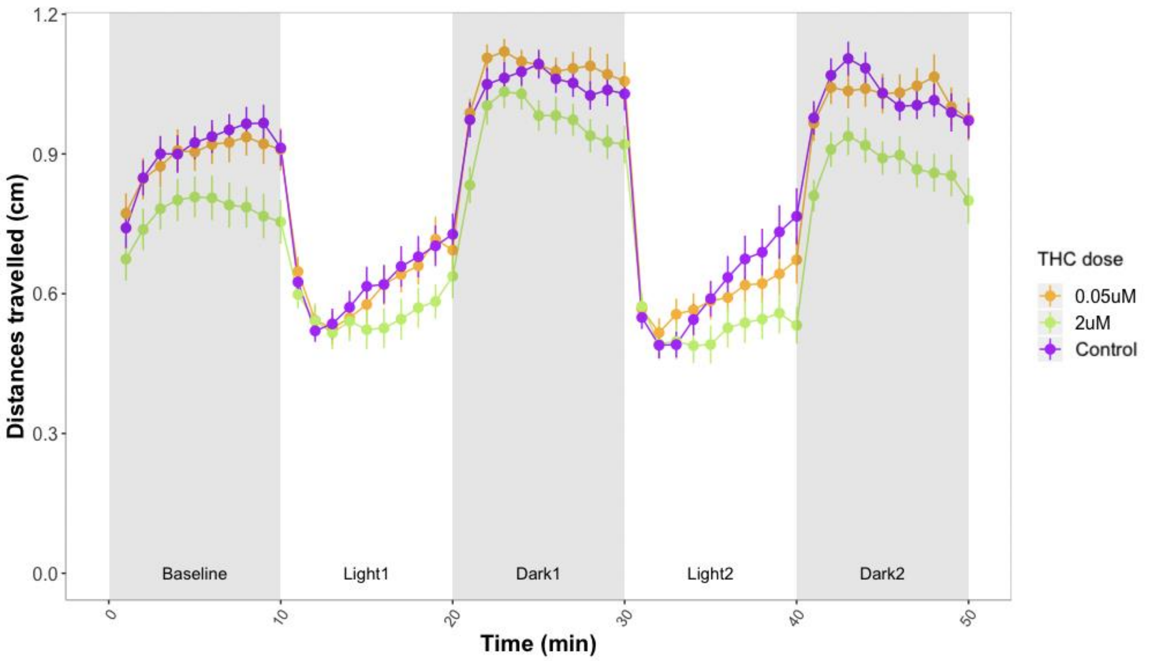

Figure S4. Forced Light-Dark test in 5 dpf wildtype zebrafish larvae after acute exposure to THC (Drug exposure: 3-4 hours prior experiment). Sample sizes n=48 per experimental group. Error bars represent standard error of the mean.

Developmental exposure to 0.15 μM nicotine from two to seven dpf led to an increase in nicotine preference when fish were adults (~four months old) and conditioned to 5 μM nicotine.

Drug-induced reinforcement of behavior, that reflects the hedonic value of drugs of abuse including nicotine, is highly conserved in both mammalian and non-mammalian species [51–54]. Conditioned place preference (CPP), where drug exposure is paired with specific environmental cues, is commonly used as a measure of drug-induced reinforcement and reward [55]. Previous studies have shown that zebrafish show a robust CPP to nicotine [32,56–58].

Here, we show developmental exposure to 0.15 μM nicotine leads to altered sensitivity of the drug-induced reinforcement and reward as measured in CPP (See [32] for methodology on the CPP assay). Fish that were not developmentally treated with nicotine showed a small increase in preference when conditioned with 5 μM nicotine. By contrast, fish exposed to 0.15 μM nicotine from two to seven days showed an increased change in preference [Interaction between CPP condition and developmental exposure: $F(1,73)=4.482$, $p=0.038$] (Figure S5).

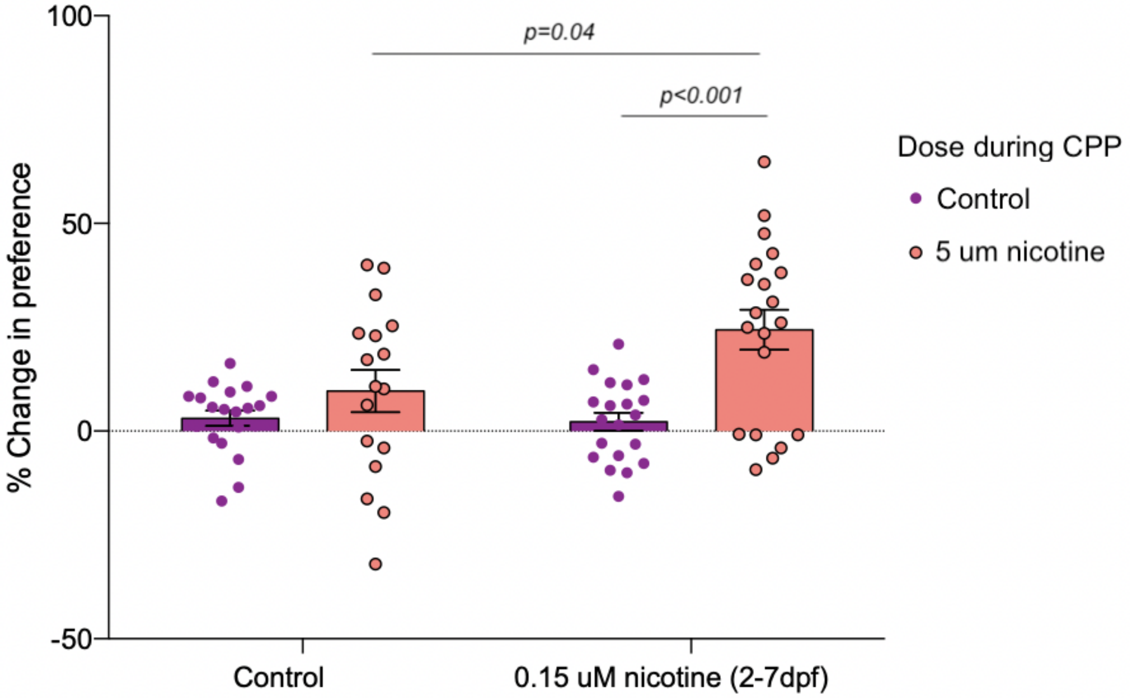

Figure S5. 5 µM nicotine-induced place preference in adult zebrafish is exacerbated by developmental exposure to 0.15 µM nicotine (from 2-7 dpf). n=17 to 20 fish per experimental group.

Appendix B: Behavioral assays data analysis

Data analysis for forced/light Dark test

Firstly, we performed an overall analysis to identify the experimental variables that were significant predictors of distance travelled during the whole duration of the experiment (50 minutes). We fitted the data to a linear mixed model with total distance travelled as response variable, experimental variables (e.g. genotype, dose, time) as fixed effects, and fish ID as random effects.

We then created three subsets of the experiment: baseline, dark, and light periods. We analyzed each subset separately by fitting the data to linear mixed models as previously described. To assess differences between the first and second light periods, and between the first and second dark periods, we added the period number as fixed effect in the linear mixed models.

Linear mixed models were calculated using the R package lme4 [59]. To identify significant fixed effects, we calculated Analysis of Deviance Tables (Type II Wald $\chi 2$ tests) for the models using the R package `car' [60]. Where significant differences were established, we carried out post-hoc Tukey tests with the R package `emmeans' [61] to further characterize the effects.

Larvae usually increased the distance travelled during the course of the light periods, and decreased it during the course of dark periods. To further explore these behaviors, we calculated linear models for each zebrafish at each light and dark period using distance travelled as response variable and time as independent variable. In these linear models, the

β coefficient for time represents the increase (or decrease) in distance travelled over time, and can be interpreted as the larva `recovery rate' from dark-light, or light-dark transitions. We constructed ANOVA models (R function `aov') to assess what variables were significant predictors of such slopes.

Data analysis for Habituation to startle response

We firstly investigated larvae spontaneous locomotion by testing whether distances travelled before the stimuli differed across experimental groups. We then investigated larvae startle responses by testing whether distances travelled during the stimuli differed across experimental groups. In both analyses, we fitted the data to linear mixed models using the R package lme4 [59], with total distance travelled as response variable, experimental variables (e.g. genotype, dose, time) as fixed effects, and fish ID as random effects.

Data analysis for novel tank diving

To analyze genotype and/or treatment differences in the *time that zebrafish spent on the bottom* of the tank, we performed beta regressions using the R package `betareg' [62]. We used beta regression because proportion time spent on the bottom of the tank was used as response variable. Proportion data is bounded by the interval [0, 1] and often exhibits heterogeneity in variance, which violates statistical assumptions used by linear models [62].

To analyze genotype or treatment differences in the *total distance* that zebrafish travelled in the tank, we fitted the data to a linear mixed model with the total distance travelled during one minute as response variable, time, genotype and/or treatment as fixed effects, and fish ID as random effects.

To analyze genotype or treatment differences in the *number of transitions* that zebrafish made between the top and the bottom of the tank, we fitted the data to a generalized linear mixed model with Poisson distribution. The Poisson distributions is commonly used when the response variable is count data [63]. We used the number of transitions to the top-bottom of the tank response variable, time, genotype or/and treatment as fixed effects, and fish ID as random effects.

Experiments were replicated on different days, and data was jointly analyzed afterwards. Mixed models were calculated using the R package lme4 [59]. To identify experimental variables with significant effects, we calculated Analysis of Deviance Tables (Type II Wald $\chi 2$ tests) for the models using the R package `car' [60]. Where significant differences were established, we carried out post-hoc Tukey tests with the R package `emmeans' [61] to further characterize the effects.

Appendix C: Adult behavior after developmental exposure to nicotine and THC in wild zebrafish

Developmental exposure to 2μM THC and 0.15 μM did not affect the time spent on the bottom of the tank, the distance travelled nor the number of transitions between the top and bottom area of the tank for wild type zebrafish (Figure S6) ($p>0.05$).

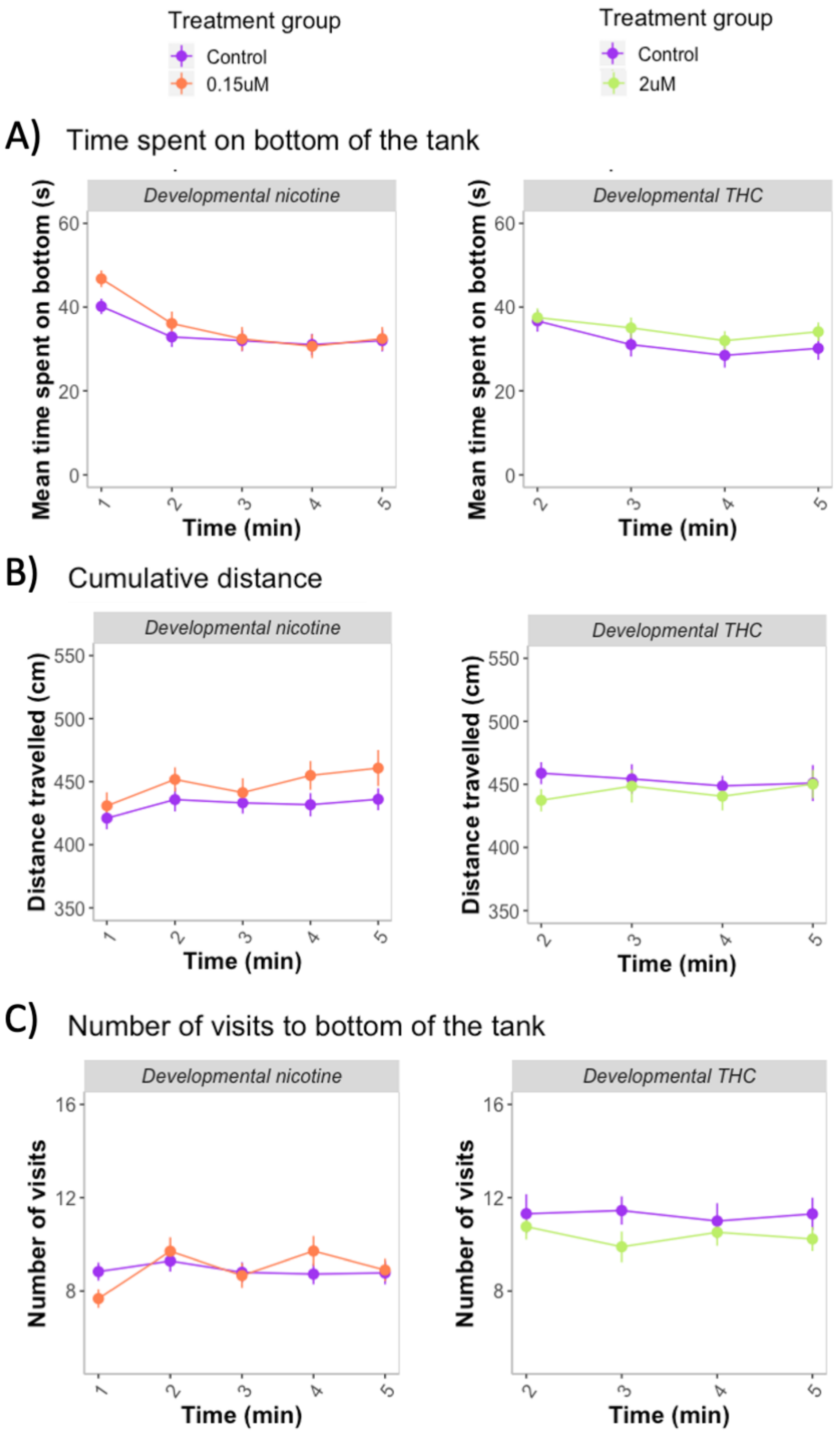

Figure S6. Novel tank diving response in adult wild type zebrafish after developmental exposure to 2μM THC and 0.15 μM nicotine. Sample sizes for each group: control THC: n=30, developmentally exposed to THC: n=40, control nicotine: n=79, developmentally exposed to nicotine: n=53. Error bars represent ±SEM.

References

1. Castaneto, M.S.; Gorelick, D.A.; Desrosiers, N.A.; Hartman, R.L.; Pirard, S.; Huestis, M.A. Synthetic Cannabinoids: Epidemiology, Pharmacodynamics, and Clinical Implications. Drug & Alcohol Dependence 2014, 144, 12–41, doi:10.1016/j.drugalcdep.2014.08.005.
2. Loeffler, G.; Delaney, E.; Hann, M. International Trends in Spice Use: Prevalence, Motivation for Use, Relationship to Other Substances, and Perception of Use and Safety for Synthetic Cannabinoids. Brain Res. Bull. 2016, 126, 8–28, doi:10.1016/j.brainresbull.2016.04.013.
3. Young-Wolff, K.C.; Tucker, L.-Y.; Alexeeff, S.; Armstrong, M.A.; Conway, A.; Weisner, C.; Goler, N. Trends in Self-Reported and Biochemically Tested Marijuana Use Among Pregnant Females in California From 2009-2016. JAMA 2017, 318, 2490–2491, doi:10.1001/jama.2017.17225.
4. Fride, E. The Endocannabinoid-CB Receptor System: Importance for Development and in Pediatric Disease. Neuro Endocrinol. Lett. 2004, 25, 24–30.
5. Jaques, S.C.; Kingsbury, A.; Henshcke, P.; Chomchai, C.; Clews, S.; Falconer, J.; Abdel-Latif, M.E.; Feller, J.M.; Oei, J.L. Cannabis, the Pregnant Woman and Her Child: Weeding out the Myths. J Perinatol 2014, 34, 417–424, doi:10.1038/jp.2013.180.
6. Järbe, T.U.C.; Deng, H.; Vadivel, S.K.; Makriyannis, A. Cannabinergic Aminoalkylindoles, Including AM678=JWH018 Found in "Spice", Examined Using Drug (Δ(9)-Tetrahydrocannabinol) Discrimination for Rats. Behav Pharmacol 2011, 22, 498–507, doi:10.1097/FBP.0b013e328349fbd5.
7. Ginsburg, B.C.; Schulze, D.R.; Hruba, L.; McMahon, L.R. JWH-018 and JWH-073: Δ9-Tetrahydrocannabinol-Like Discriminative Stimulus Effects in Monkeys. J Pharmacol Exp Ther 2012, 340, 37–45, doi:10.1124/jpet.111.187757.
8. Tait, R.J.; Caldicott, D.; Mountain, D.; Hill, S.L.; Lenton, S. A Systematic Review of Adverse Events Arising from the Use of Synthetic Cannabinoids and Their Associated Treatment. Clinical Toxicology 2016, 54, 1–13, doi:10.3109/15563650.2015.1110590.
9. Theunissen, E.L.; Hutten, N.R.P.W.; Mason, N.L.; Toennes, S.W.; Kuypers, K.P.C.; de Sousa Fernandes Perna, E.B.; Ramaekers, J.G. Neurocognition and Subjective Experience Following Acute Doses of the Synthetic Cannabinoid JWH-018: A Phase 1, Placebo-Controlled, Pilot Study. Br. J. Pharmacol. 2018, 175, 18–28, doi:10.1111/bph.14066.
10. Carlier, J.; Huestis, M.A.; Zaami, S.; Pichini, S.; Busardò, F.P. Monitoring the Effects of Perinatal Cannabis and Synthetic Cannabinoid Exposure. Therapeutic Drug Monitoring 2019, Publish Ahead of Print, doi:10.1097/FTD.0000000000000667.
11. Brandon, N.J.; Sawa, A. Linking Neurodevelopmental and Synaptic Theories of Mental Illness through DISC1. Nat. Rev. Neurosci. 2011, 12, 707–722, doi:10.1038/nrn3120.
12. Jacobs, P.A.; Brunton, M.; Frackiewicz, A.; Newton, M.; Cook, P.J.L.; Robson, E.B. Studies on a Family with Three Cytogenetic Markers. Annals of Human Genetics 1970, 33, 325–336, doi:10.1111/j.1469-1809.1970.tb01658.x.
13. Blackwood, D.H.R.; Fordyce, A.; Walker, M.T.; St. Clair, D.M.; Porteous, D.J.; Muir, W.J. Schizophrenia and Affective Disorders—Cosegregation with a Translocation at Chromosome 1q42 That Directly Disrupts Brain-Expressed Genes: Clinical and P300 Findings in a Family. Am J Hum Genet 2001, 69, 428–433.
14. Ballinger, M.D.; Saito, A.; Abazyan, B.; Taniguchi, Y.; Huang, C.-H.; Ito, K.; Zhu, X.; Segal, H.; Jaaro-Peled, H.; Sawa, A.; et al. Adolescent Cannabis Exposure Interacts with Mutant DISC1 to Produce Impaired Adult Emotional Memory. Neurobiol Dis 2015, 82, 176–184, doi:10.1016/j.nbd.2015.06.006.
15. Jouroukhin, Y.; Zhu, X.; Shevelkin, A.V.; Hasegawa, Y.; Abazyan, B.; Saito, A.; Pevsner, J.; Kamiya, A.; Pletnikov, M.V. Adolescent Δ9-Tetrahydrocannabinol Exposure and Astrocyte-Specific Genetic Vulnerability Converge on Nuclear Factor-KB-Cyclooxygenase-2 Signaling to Impair Memory in Adulthood. Biol. Psychiatry 2019, 85, 891–903, doi:10.1016/j.biopsych.2018.07.024.
16. Clark, K.J.; Boczek, N.J.; Ekker, S.C. Stressing Zebrafish for Behavioral Genetics. Rev Neurosci 2011, 22, 49–62, doi:10.1515/RNS.2011.007.
17. Egan, R.J.; Bergner, C.L.; Hart, P.C.; Cachat, J.M.; Canavello, P.R.; Elegante, M.F.; Elkhayat, S.I.; Bartels, B.K.; Tien, A.K.; Tien, D.H.; et al. Understanding Behavioral and Physiological Phenotypes of Stress and Anxiety in Zebrafish. Behav Brain Res 2009, 205, 38–44, doi:10.1016/j.bbr.2009.06.022.
18. Bossé, G.D.; Peterson, R.T. Development of an Opioid Self-Administration Assay to Study Drug Seeking in Zebrafish. Behavioural Brain Research 2017, 335, 158–166, doi:10.1016/j.bbr.2017.08.001.

19. Iacono, W.G. Endophenotypes in Psychiatric Disease: Prospects and Challenges. Genome Med 2018, 10, doi:10.1186/s13073-018-0526-5.
20. De Rienzo, G.; Bishop, J.A.; Mao, Y.; Pan, L.; Ma, T.P.; Moens, C.B.; Tsai, L.-H.; Sive, H. Disc1 Regulates Both β-Catenin-Mediated and Noncanonical Wnt Signaling during Vertebrate Embryogenesis. The FASEB Journal 2011, 25, 4184–4197, doi:10.1096/fj.11-186239.
21. Henn, K.; Braunbeck, T. Dechorionation as a Tool to Improve the Fish Embryo Toxicity Test (FET) with the Zebrafish (Danio Rerio). Comparative Biochemistry and Physiology Part C: Toxicology & Pharmacology 2011, 153, 91–98, doi:10.1016/j.cbpc.2010.09.003.
22. Kais, B.; Schneider, K.E.; Keiter, S.; Henn, K.; Ackermann, C.; Braunbeck, T. DMSO Modifies the Permeability of the Zebrafish (Danio Rerio) Chorion-Implications for the Fish Embryo Test (FET). Aquatic Toxicology 2013, 140–141, 229–238, doi:10.1016/j.aquatox.2013.05.022.
23. Achenbach, J.C.; Hill, J.; Hui, J.P.M.; Morash, M.G.; Berrue, F.; Ellis, L.D. Analysis of the Uptake, Metabolism, and Behavioral Effects of Cannabinoids on Zebrafish Larvae. Zebrafish 2018, 15, 349–360, doi:10.1089/zeb.2017.1541.
24. De Luca, M.A.; Bimpisidis, Z.; Melis, M.; Marti, M.; Caboni, P.; Valentini, V.; Margiani, G.; Pintori, N.; Polis, I.; Marsicano, G.; et al. Stimulation of in Vivo Dopamine Transmission and Intravenous Self-Administration in Rats and Mice by JWH-018, a Spice Cannabinoid. Neuropharmacology 2015, 99, 705–714, doi:10.1016/j.neuropharm.2015.08.041.
25. Elmore, J.S.; Baumann, M.H. Repeated Exposure to the "Spice" Cannabinoid JWH-018 Induces Tolerance and Enhances Responsiveness to 5-HT1A Receptor Stimulation in Male Rats. Front Psychiatry 2018, 9, doi:10.3389/fpsyt.2018.00055.
26. Irons, T.D.; MacPhail, R.C.; Hunter, D.L.; Padilla, S. Acute Neuroactive Drug Exposures Alter Locomotor Activity in Larval Zebrafish. Neurotoxicol Teratol 2010, 32, 84–90, doi:10.1016/j.ntt.2009.04.066.
27. Glazer, L.; Hawkey, A.B.; Wells, C.N.; Drastal, M.; Odamah, K.-A.; Behl, M.; Levin, E.D. Developmental Exposure to Low Concentrations of Organophosphate Flame Retardants Causes Life-Long Behavioral Alterations in Zebrafish. Toxicol Sci 2018, 165, 487–498, doi:10.1093/toxsci/kfy173.
28. Burgess, H.A.; Granato, M. Sensorimotor Gating in Larval Zebrafish. J. Neurosci. 2007, 27, 4984–4994, doi:10.1523/JNEUROSCI.0615-07.2007.
29. Rankin, C.H.; Abrams, T.; Barry, R.J.; Bhatnagar, S.; Clayton, D.; Colombo, J.; Coppola, G.; Geyer, M.A.; Glanzman, D.L.; Marsland, S.; et al. Habituation Revisited: An Updated and Revised Description of the Behavioral Characteristics of Habituation. Neurobiol Learn Mem 2009, 92, 135–138, doi:10.1016/j.nlm.2008.09.012.
30. Quednow, B.B.; Wagner, M.; Westheide, J.; Beckmann, K.; Bliesener, N.; Maier, W.; Kühn, K.-U. Sensorimotor Gating and Habituation of the Startle Response in Schizophrenic Patients Randomly Treated with Amisulpride or Olanzapine. Biol. Psychiatry 2006, 59, 536–545, doi:10.1016/j.biopsych.2005.07.012.
31. Best, J.D.; Berghmans, S.; Hunt, J.J.F.G.; Clarke, S.C.; Fleming, A.; Goldsmith, P.; Roach, A.G. Non-Associative Learning in Larval Zebrafish. Neuropsychopharmacology 2008, 33, 1206–1215, doi:10.1038/sj.npp.1301489.
32. García-González, J.; Brock, A.J.; Parker, M.O.; Riley, R.J.; Joliffe, D.; Sudwarts, A.; Teh, M.-T.; Busch-Nentwich, E.M.; Stemple, D.L.; Martineau, A.R.; et al. Identification of Slit3 as a Locus Affecting Nicotine Preference in Zebrafish and Human Smoking Behaviour. Elife 2020, 9, doi:10.7554/eLife.51295.
33. Parker, M.O.; Millington, M.E.; Combe, F.J.; Brennan, C.H. Housing Conditions Differentially Affect Physiological and Behavioural Stress Responses of Zebrafish, as Well as the Response to Anxiolytics. PLoS ONE 2012, 7, e34992, doi:10.1371/journal.pone.0034992.
34. Basnet, R.M.; Zizioli, D.; Taweedet, S.; Finazzi, D.; Memo, M. Zebrafish Larvae as a Behavioral Model in Neuropharmacology. Biomedicines 2019, 7, doi:10.3390/biomedicines7010023.
35. Petzold, A.M.; Balciunas, D.; Sivasubbu, S.; Clark, K.J.; Bedell, V.M.; Westcot, S.E.; Myers, S.R.; Moulder, G.L.; Thomas, M.J.; Ekker, S.C. Nicotine Response Genetics in the Zebrafish. PNAS 2009, doi:10.1073/pnas.0908247106.
36. Ahmed, K.T.; Amin, M.R.; Shah, P.; Ali, D.W. Motor Neuron Development in Zebrafish Is Altered by Brief (5-Hr) Exposures to THC (Δ 9 -Tetrahydrocannabinol) or CBD (Cannabidiol) during Gastrulation. Sci Rep 2018, 8, 1–14, doi:10.1038/s41598-018-28689-z.
37. Lee, H.B.; Schwab, T.L.; Sigafoos, A.N.; Gauerke, J.L.; Krug, R.G.; Serres, M.R.; Jacobs, D.C.; Cotter, R.P.; Das, B.; Petersen, M.O.; et al. Novel Zebrafish Behavioral Assay to Identify Modifiers of the Rapid, Nongenomic Stress Response. Genes Brain Behav 2019, 18, doi:10.1111/gbb.12549.

38. Am, S.; Av, K. The Behavioral Effects of Acute Δ9-Tetrahydrocannabinol and Heroin (Diacetylmorphine) Exposure in Adult Zebrafish. Brain Res 2013, 1543, 109–119, doi:10.1016/j.brainres.2013.11.002.
39. Levin, E.D.; Bencan, Z.; Cerutti, D.T. Anxiolytic Effects of Nicotine in Zebrafish. Physiol. Behav. 2007, 90, 54–58, doi:10.1016/j.physbeh.2006.08.026.
40. López-Patiño, M.A.; Yu, L.; Cabral, H.; Zhdanova, I.V. Anxiogenic Effects of Cocaine Withdrawal in Zebrafish. Physiol. Behav. 2008, 93, 160–171, doi:10.1016/j.physbeh.2007.08.013.
41. Krook, J.T.; Duperreault, E.; Newton, D.; Ross, M.S.; Hamilton, T.J. Repeated Ethanol Exposure Increases Anxiety-like Behaviour in Zebrafish during Withdrawal. PeerJ 2019, 7, e6551, doi:10.7717/peerj.6551.
42. Vaz, R.; Hofmeister, W.; Lindstrand, A. Zebrafish Models of Neurodevelopmental Disorders: Limitations and Benefits of Current Tools and Techniques. International Journal of Molecular Sciences 2019, 20, 1296, doi:10.3390/ijms20061296.
43. Huang, L.Z.; Liu, X.; Griffith, W.H.; Winzer-Serhan, U.H. Chronic Neonatal Nicotine Increases Anxiety but Does Not Impair Cognition in Adult Rats. Behav Neurosci 2007, 121, 1342–1352, doi:10.1037/0735-7044.121.6.1342.
44. Eddins, D.; Cerutti, D.; Williams, P.; Linney, E.; Levin, E.D. Zebrafish Provide a Sensitive Model of Persisting Neurobehavioral Effects of Developmental Chlorpyrifos Exposure: Comparison with Nicotine and Pilocarpine Effects and Relationship to Dopamine Deficits. Neurotoxicol Teratol 2010, 32, 99–108, doi:10.1016/j.ntt.2009.02.005.
45. Eachus, H.; Bright, C.; Cunliffe, V.T.; Placzek, M.; Wood, J.D.; Watt, P.J. Disrupted-in-Schizophrenia-1 Is Essential for Normal Hypothalamic-Pituitary-Interrenal (HPI) Axis Function. Hum. Mol. Genet. 2017, 26, 1992–2005, doi:10.1093/hmg/ddx076.
46. Wood, J.D.; Bonath, F.; Kumar, S.; Ross, C.A.; Cunliffe, V.T. Disrupted-in-Schizophrenia 1 and Neuregulin 1 Are Required for the Specification of Oligodendrocytes and Neurones in the Zebrafish Brain. Hum. Mol. Genet. 2009, 18, 391–404, doi:10.1093/hmg/ddn361.
47. Drerup, C.M.; Wiora, H.M.; Topczewski, J.; Morris, J.A. Disc1 Regulates Foxd3 and Sox10 Expression, Affecting Neural Crest Migration and Differentiation. Development 2009, 136, 2623–2632, doi:10.1242/dev.030577.
48. Craft, R.M. Sex Differences in Behavioral Effects of Cannabinoids. Life Sci 2005, 77, 2471–2478, doi:10.1016/j.lfs.2005.04.019.
49. Kaminitz, A.; Barzilay, R.; Segal, H.; Taler, M.; Offen, D.; Gil-Ad, I.; Mechoulam, R.; Weizman, A. Dominant Negative DISC1 Mutant Mice Display Specific Social Behaviour Deficits and Aberration in BDNF and Cannabinoid Receptor Expression. World J. Biol. Psychiatry 2014, 15, 76–82, doi:10.3109/15622975.2013.841993.
50. Ogungbemi, A.; Leuthold, D.; Scholz, S.; Küster, E. Hypo- or Hyperactivity of Zebrafish Embryos Provoked by Neuroactive Substances: A Review on How Experimental Parameters Impact the Predictability of Behavior Changes. Environmental Sciences Europe 2019, 31, 88, doi:10.1186/s12302-019-0270-5.
51. Brennan, C.H.; Parker, M.O. Zebrafish (Danio Rerio) Models of Substance Abuse: Harnessing the Capabilities. Behaviour 2012, 149, 1037–1062, doi:10.1163/1568539X-00003010.
52. Engleman, E.A.; Katner, S.N.; Neal-Beliveau, B.S. Caenorhabditis Elegans as a Model to Study the Molecular and Genetic Mechanisms of Drug Addiction. Prog Mol Biol Transl Sci 2016, 137, 229–252, doi:10.1016/bs.pmbts.2015.10.019.
53. Shipley, A.T.; Imeh-Nathaniel, A.; Orfanakos, V.B.; Wormack, L.N.; Huber, R.; Nathaniel, T.I. The Sensitivity of the Crayfish Reward System to Mammalian Drugs of Abuse. Front. Physiol. 2017, 8, doi:10.3389/fphys.2017.01007.
54. Berridge, K.C.; Kringelbach, M.L. Affective Neuroscience of Pleasure: Reward in Humans and Animals. Psychopharmacology (Berl) 2008, 199, 457–480, doi:10.1007/s00213-008-1099-6.
55. Tzschentke, T.M. Measuring Reward with the Conditioned Place Preference Paradigm: A Comprehensive Review of Drug Effects, Recent Progress and New Issues. Prog. Neurobiol. 1998, 56, 613–672.
56. Kedikian, X.; Faillace, M.P.; Bernabeu, R. Behavioral and Molecular Analysis of Nicotine-Conditioned Place Preference in Zebrafish. PLOS ONE 2013, 8, e69453, doi:10.1371/journal.pone.0069453.
57. Ponzoni, L.; Braida, D.; Pucci, L.; Andrea, D.; Fasoli, F.; Manfredi, I.; Papke, R.L.; Stokes, C.; Cannazza, G.; Clementi, F.; et al. The Cytisine Derivatives, CC4 and CC26, Reduce Nicotine-Induced Conditioned Place Preference in Zebrafish by Acting on Heteromeric Neuronal Nicotinic Acetylcholine Receptors. Psychopharmacology (Berl.) 2014, 231, 4681–4693, doi:10.1007/s00213-014-3619-x.

58. Brock, A.J.; Goody, S.M.G.; Mead, A.N.; Sudwarts, A.; Parker, M.O.; Brennan, C.H. Assessing the Value of the Zebrafish Conditioned Place Preference Model for Predicting Human Abuse Potential. J. Pharmacol. Exp. Ther. 2017, 363, 66–79, doi:10.1124/jpet.117.242628.
59. Bates, D.; Mächler, M.; Bolker, B.; Walker, S. Fitting Linear Mixed-Effects Models Using Lme4. Journal of Statistical Software 2015, 67, 1–48, doi:10.18637/jss.v067.i01.
60. Fox, J.; Weisberg, S.; Price, B.; Adler, D.; Bates, D.; Baud-Bovy, G.; Bolker, B.; Ellison, S.; Firth, D.; Friendly, M.; et al. Car: Companion to Applied Regression; 2019;
61. Lenth, R.; Singmann, H.; Love, J.; Buerkner, P.; Herve, M. Emmeans: Estimated Marginal Means, Aka Least-Squares Means; 2019;
62. Zeileis, A.; Cribari-Neto, F.; Gruen, B.; Kosmidis, I.; by), A.B.S. (earlier version; by), A.V.R. (earlier version Betareg: Beta Regression; 2019;
63. Plan, E.L. Modeling and Simulation of Count Data. CPT Pharmacometrics Syst Pharmacol 2014, 3, e129, doi:10.1038/psp.2014.27.

021 by the author. Licensee MDPI, Basel, Switzerland. This article is an open access article distributed under the terms and conditions of the Creative Commons Attribution (CC BY) license (http://creativecommons.org/licenses/by/4.0/).
